# Antioxidant Strategies in Testicular Ischemia–Reperfusion Injury—Translational Insights and Clinical Implications for Testicular Torsion Management

**DOI:** 10.3390/antiox15081029

**Published:** 2026-08-18

**Authors:** Marko Bašković, Davor Ježek

**Affiliations:** 1 School of Medicine, Catholic University of Croatia, Ilica 242, 10000 Zagreb, Croatia; 2 Department of Pediatric Surgery, Children’s Hospital Zagreb, Ulica Vjekoslava Klaića 16, 10000 Zagreb, Croatia; 3 School of Medicine, University of Zagreb, Šalata 3, 10000 Zagreb, Croatia; davor.jezek@mef.hr; 4 Scientific Centre of Excellence for Reproductive and Regenerative Medicine, School of Medicine, University of Zagreb, Šalata 3, 10000 Zagreb, Croatia; 5 Croatian Academy of Medical Sciences, Kaptol 15, 10000 Zagreb, Croatia; 6 Department of Histology and Embryology, School of Medicine, University of Zagreb, Šalata 3, 10000 Zagreb, Croatia; 7 Department of Transfusion Medicine and Transplantation Biology, University Hospital Centre Zagreb, Kišpatićeva 12, 10000 Zagreb, Croatia

**Keywords:** testicular torsion, ischemia–reperfusion injury, oxidative stress, antioxidants, reactive oxygen species, germ cell apoptosis, male infertility, translational medicine, clinical trial design, spermatic cord detorsion

## Abstract

Testicular torsion is a time-critical urological emergency in which surgical detorsion, the only accepted treatment, simultaneously rescues and injures the gonad. Restoration of blood flow triggers a burst of reactive oxygen and nitrogen species, neutrophil recruitment, inflammasome activation, and regulated germ cell death, so that a substantial proportion of anatomically salvaged testes still undergo atrophy and functional loss. A large experimental literature, running to several hundred reports, has shown that antioxidants of almost every chemical class attenuate this injury in rodent models, yet no antioxidant has entered routine clinical use as an adjunct to detorsion in humans, and we are not aware of any adequately powered randomized trial in this setting. This narrative review integrates the redox pathophysiology of testicular ischemia–reperfusion injury with an appraisal of the agents tested against it, and then asks why the translational gap has proved so durable. We examine model heterogeneity, the dominance of pretreatment designs that cannot be reproduced in an emergency department, the reliance on short-term surrogate biochemistry rather than fertility endpoints, and the sobering precedents of neuroprotection and cardioprotection. We close with a roadmap for first-in-human evaluation, covering candidate prioritization, route and timing of administration, biomarker selection, and a feasible trial architecture.

## 1. Introduction

Torsion of the spermatic cord is one of the very few conditions in which the reproductive future of an otherwise healthy young man can be decided within the space of a single afternoon. It accounts for a large share of presentations of the acute scrotum in boys and adolescents [1,2], the annual incidence is conventionally quoted as approximately one case per four thousand males below twenty-five years of age, and the age distribution is bimodal with peaks in the neonatal period and around puberty [3,4]. The diagnosis remains fundamentally clinical, supported where necessary by color Doppler ultrasonography and by structured scoring instruments [5,6]. The treatment has not changed in its essentials for more than a century. The cord is untwisted, the testis is inspected, and the organ is either fixed in place or removed.

That simplicity is deceptive. Surgical detorsion corrects an anatomical problem, and it does so reliably, but it does not correct the biology that the torsion has set in motion. Outcome after torsion is governed above all by the interval between the onset of pain and the restoration of perfusion, and by the number of turns of the cord [7]. Series from high-income and low-income settings alike continue to report orchiectomy in a substantial minority of explored patients, driven almost entirely by late presentation rather than by any deficiency of surgical technique [4,8]. What is less widely appreciated in everyday practice is that anatomical salvage is not the same as functional salvage. A testis that looks pink in the operating theater and is duly fixed to the dartos may still atrophy over the following months, and long-term follow-up studies of men treated for torsion in childhood or adolescence document reduced testicular volume, altered gonadotropin profiles and impaired semen quality in a meaningful fraction of patients [9,10].

The reason lies in a paradox that is familiar from every other organ subject to acute vascular occlusion. Reperfusion is indispensable, and reperfusion is injurious. During the ischemic phase, adenosine triphosphate is consumed, ionic gradients collapse, intracellular calcium rises, and xanthine dehydrogenase is proteolytically converted to xanthine oxidase while its substrate hypoxanthine accumulates. The moment molecular oxygen returns, that primed enzymatic machinery, together with mitochondrial reverse electron transport, activated nicotinamide adenine dinucleotide phosphate oxidases and uncoupled nitric oxide synthase, generates a burst of reactive oxygen and reactive nitrogen species that far exceeds the buffering capacity of the tissue [11,12,13]. The testis is an unusually poor host for such an event. It operates at a low physiological oxygen tension, its germinal epithelium is rich in polyunsaturated fatty acids that are exquisite substrates for lipid peroxidation, its mitotic and meiotic activity is among the highest in the body, and much of its antioxidant reserve is compartmentalized behind the blood–testis barrier [14,15]. Experimental work in the rat and the mouse established more than two decades ago that ischemia followed by reperfusion produces a germ cell-specific wave of apoptosis that peaks approximately one day after the repair of torsion, and that this wave depends on the recruitment of neutrophils to subtunical venules rather than on the ischemic insult alone [16,17,18].

If reperfusion injury is the mechanism, then pharmacological interruption of that mechanism at the moment of detorsion is an obvious therapeutic idea, and the experimental community has pursued it with remarkable energy. Vitamins, carotenoids, thiol donors, indoleamines, hundreds of plant-derived polyphenols, enzyme inhibitors, anesthetic agents, calcium channel blockers, phosphodiesterase inhibitors, gasotransmitters, hyperbaric oxygen, ischemic conditioning protocols, mesenchymal stromal cells and their exosomes have all been tested in torsion–detorsion models, and the overwhelming majority of these studies report a benefit [11,15,19,20]. Reviews of the field regularly catalog dozens of effective molecules and conclude, quite reasonably, that adjuvant antioxidant therapy is promising.

And yet none of this has reached the bedside. There is no antioxidant, and no other cytoprotective agent, that is administered as standard during or after surgical detorsion of the testis in any guideline or standard of care that we have been able to identify. There is no product license for such an indication. There is, to the best of our knowledge, no adequately powered randomized controlled trial of any antioxidant given at the time of detorsion with a fertility-relevant primary endpoint. The adjacent field of male subfertility offers a warning, although a narrow one. There, oral antioxidants have been trialed extensively and the accumulated evidence remains of low to very low certainty, with the largest well-conducted multicenter trial finding no effect on semen parameters, sperm deoxyribonucleic acid integrity, pregnancy or live birth [21,22]. That experience does not test the acute hypothesis, because chronic oral supplementation in a man with idiopathic oligozoospermia is a different intervention from a single parenteral dose given at a defined reperfusion event; we return to this distinction in Section 7.2. What it does show is that a large body of small positive trials can coexist with an absence of benefit once an adequately powered study is finally performed, which is the specific failure mode with which this review is concerned. A therapeutic idea that has succeeded in rodent models on almost every occasion on which it has been tested, and that has never once been tested properly in humans, is not a promising idea. It is an unexamined one.

This review takes that observation as its organizing question. We first set out the clinical anatomy of the problem, including what surgery does and does not achieve and what is known about long-term function after salvage. We then develop the redox and inflammatory pathophysiology of testicular ischemia–reperfusion injury in the detail required to evaluate the drugs aimed at it, including the temporal sequencing of the different sources of oxidant stress and the several forms of regulated cell death now implicated. The core of the article is a systematic agent-by-agent appraisal of the antioxidant armamentarium, organized by mechanism and summarized in tabular form, in which we record not only whether an agent worked but when it was given, in what species, at what torsion degree and duration, and against which endpoints. We then turn the analysis around and ask what features of this literature predict translational failure, drawing explicitly on the experience of neuroprotection in acute stroke and cardioprotection in myocardial infarction, where a comparable abundance of positive preclinical data produced almost no clinical benefit. Finally, we propose a concrete roadmap, including criteria for candidate selection, the pharmacological constraints imposed by an emergency surgical pathway, a biomarker strategy, and a trial architecture that a pediatric surgical network could realistically deliver. This is a narrative rather than a systematic review, and the literature underlying it was identified by searching PubMed, MEDLINE, Scopus, Web of Science, and Google Scholar from inception to July 2026, using combinations of the terms testicular torsion, testis, ischemia–reperfusion, oxidative stress, and antioxidant.

## 2. The Clinical Problem and the Reperfusion Window

### 2.1. Epidemiology and the Arithmetic of Delay

Torsion is uncommon in absolute terms and common in the population that matters most for future fertility. The frequently cited figure of roughly one case per four thousand males below twenty-five years of age comes from older hospital-based series, and modern administrative datasets give incidence estimates that vary with case definition and health system, but every dataset reproduces the same bimodal age structure and the same striking concentration of cases in the peripubertal years [1,3,4]. Because the condition is uncommon at the level of the individual clinician and dramatic at the level of the individual patient, it generates a characteristic pattern of system failure. Boys delay reporting scrotal pain out of embarrassment, primary care and emergency physicians confuse the presentation with epididymo-orchitis, and inter-hospital transfer consumes the remaining time [5,8].

The consequence is that the single most powerful determinant of outcome is not any property of the drug cabinet but the clock. Multivariate analyses consistently identify symptom duration as the dominant predictor of testicular salvage, with the degree of cord twisting contributing independently, so that a testis twisted through more than a full turn and explored late is very unlikely to survive [7]. Reported orchiectomy rates in explored patients differ substantially between health systems and between case definitions, with a figure of about forty percent in boys undergoing surgery being widely quoted [5], and with national datasets returning both appreciably lower and appreciably higher estimates depending on how cases are ascertained [4,8,23]. Whatever the local figure, it has proved stubbornly resistant to public awareness campaigns and to emergency department triage protocols. It is worth stating plainly what this means for the present review. Any pharmacological adjunct will be given to a population in which the primary injury has already been determined by factors entirely outside the surgeon’s control, and in which the therapeutic question is therefore not whether a necrotic testis can be resurrected but whether a marginally viable one can be preserved in better functional condition than it would otherwise have been. It follows that the largest available gain in this disease is not pharmacological at all. It lies in shortening the interval between the onset of pain and the restoration of flow, and an adjuvant drug can only act on the residual injury that remains once that interval has been fixed. We regard the two objectives as complementary rather than competing, but the asymmetry between their likely effect sizes should be kept in mind throughout what follows, and it is one of the reasons why we argue later for a cheap and pragmatic first trial rather than an expensive one.

### 2.2. What the Operation Solves and What It Does Not

Scrotal exploration with detorsion and bilateral orchiopexy is an excellent operation. It relieves the mechanical obstruction, it permits direct assessment of viability, and by fixing the contralateral gonad it removes the substantial risk conferred by the bell clapper anomaly, which is usually bilateral. What the operation cannot do is neutralize the biochemical consequences of the ischemic interval that preceded it. On the contrary, the untwisting of the cord is the precise event that initiates reperfusion injury. The surgeon who restores arterial inflow to a testis that has been anoxic for eight hours is delivering a bolus of molecular oxygen to a tissue whose xanthine oxidase has been primed, whose mitochondrial electron transport chain is in a reduced state, whose intracellular calcium is elevated and whose endothelium is already expressing adhesion molecules [11,12,13].

This gives testicular torsion an unusual and, for the purposes of trial design, an extremely favorable feature. In acute ischemic stroke and in myocardial infarction the moment of reperfusion is either unwitnessed or spread over a variable interval, and the pharmacological window has to be defined by proxy. In torsion the moment of reperfusion is created deliberately, by a named individual, in a controlled environment, at a documented time. A cytoprotective agent could in principle be administered intravenously at induction of anesthesia, or infused locally into the spermatic cord, or applied topically to the tunica, in each case with the reperfusion event timed to the intervention rather than the other way round. Very few clinical situations offer this degree of control, and it is one of the reasons why the complete absence of human trials in this field is so difficult to justify [13,19,20].

One qualification must be entered at once. Not every patient reaches the operating theater with the cord still twisted. Attempted manual detorsion in the emergency department is practised in many centers and has been associated with improved rates of testicular salvage at exploration [24], and although it remains infrequently performed in pediatric emergency practice, particularly where urological cover is available [25], it is common enough that a trial cannot ignore it. Where the maneuver succeeds, wholly or in part, reperfusion begins earlier, outside the operating theater, unwitnessed and untimed. That weakens the premise of a single deliberate reperfusion event, it confounds symptom duration as a prognostic and stratification variable, and it means that any protocol built on the argument above must record whether manual detorsion was attempted and whether it was judged successful, and must stratify or exclude accordingly.

### 2.3. Function of the Anatomically Salvaged Testis

The clinical literature has historically used orchiectomy as the outcome of record, which is convenient and misleading. A testis that is preserved is not necessarily a testis that works. Follow-up series document late atrophy in a considerable proportion of organs that appeared viable at exploration, and the functional data are more sobering still. Population-based work has shown that men with a history of testicular torsion have measurably altered reproductive parameters relative to unaffected peers, including lower testicular volume and shifts in gonadotropin and inhibin B concentrations consistent with reduced Sertoli cell mass [9]. Prospective long-term evaluation of men operated for suspected torsion has similarly identified impaired semen quality, elevated seminal oxidative stress and reduced testicular volume on the affected side [10]. Reviews of the whole body of outcome data conclude that a clinically meaningful minority of patients are left with subnormal spermatogenesis even after timely and technically successful surgery [26].

Not all of the evidence points in one direction, and it is important to be fair to it. Studies with paternity as the endpoint have generally been more reassuring than studies with semen analysis as the endpoint, which is what one would expect given the large functional reserve of the male reproductive system and the fact that most men father children with sperm counts well below the laboratory optimum [27]. The classic observation of abnormal semen quality after torsion in the pre-ultrasound era was made in small cohorts with limited control for confounding [28]. Nevertheless, the direction of effect is consistent across four decades and across species. Experimental torsion in the rat reduces sperm output and alters both endocrine and exocrine testicular function, with the magnitude of the deficit scaling with the duration and the angle of torsion, and with the deficit persisting long after the acute event has resolved [29,30,31]. The reasonable summary is that anatomical salvage protects a great deal but not everything, and that the residual deficit is precisely the target that an adjuvant antioxidant would be expected to address.

### 2.4. The Contralateral Testis

No discussion of torsion can avoid the long-running argument about the untorted gonad. The earliest experimental answer was negative, since acute unilateral torsion in the rat was reported to leave the contralateral testis unaffected [32], and the fertility consequences of prepubertal cord torsion in the same species were characterized in the same period [33]. Later work reached the opposite conclusion. Several groups have reported that unilateral torsion produces histological and biochemical abnormalities in the contralateral testis, including germ cell apoptosis, neutrophil recruitment and endothelial E-selectin expression, and this has been interpreted variously as an autoimmune phenomenon triggered by breach of the blood–testis barrier, as a sympathetically mediated reflex vasoconstriction, and as a systemic oxidative event. Measurements of contralateral intratesticular biochemistry after unilateral cord torsion have supported the presence of hypoxia-like changes on the opposite side [34], and contralateral blood flow has been reported to fall during ipsilateral torsion [35]. The strongest experimental statement of the case remains the demonstration that ipsilateral ischemia and reperfusion is followed by neutrophil recruitment, adhesion molecule expression and germ cell apoptosis in the contralateral organ [36].

Against this stands the negative evidence already cited above [32], which came from a laboratory central to the definition of the ipsilateral injury, together with the general objection that a contralateral effect becomes difficult to demonstrate once appropriate sham controls are used and once the confounding influence of surgical manipulation itself is removed. Our own view is that the contralateral question is not settled, that the effect if real is small relative to the ipsilateral injury, and that it should be treated in trial design as a secondary rather than a primary consideration. It matters for the present argument mainly because it changes the theoretical ceiling of benefit. If a systemically administered antioxidant protects both gonads, the effect size available to a trial is larger than if it protects only the organ that was twisted. It also has a direct consequence for outcome measurement that is easy to overlook. If the contralateral testis is itself injured, or is itself protected by a systemically administered agent, then any endpoint expressed as a ratio of the affected to the unaffected side is insensitive by construction, because injury or protection of the denominator moves the ratio towards unity. Since bilateral orchiopexy means that the opposite gonad is surgically handled in every patient as well, we argue in Section 9.3 for absolute rather than relative measurement of testicular volume.

### 2.5. Ischemia Duration and the Magnitude of the Reperfusion Injury

The clinical literature answers the salvage question with reasonable precision and the reperfusion question only indirectly; the distinction matters for anyone deciding how long a pharmacological window stays open. Pooled case series describe a graded rather than a threshold relationship. In the largest systematic synthesis of time to treatment, covering 2116 patients, testicular survival was 97.2 percent when detorsion occurred within six hours of symptom onset, 79.3 percent between seven and twelve hours, 61.3 percent between thirteen and eighteen hours, 42.5 percent between nineteen and twenty-four hours, 24.4 percent between twenty-five and forty-eight hours and 7.4 percent beyond forty-eight hours [37]. Expressed cumulatively, the same dataset gives 90.4 percent salvage within twelve hours, 54.0 percent between thirteen and twenty-four hours and 18.1 percent beyond twenty-four hours [37]. Duration of symptoms has been reported as the only independent predictor of salvage in a case–control analysis [38], and it remains the dominant predictor in almost every series. The practical reading is that no single hour marks the loss of the organ. Risk accumulates continuously from the first hour, it rises steeply between six and twelve hours, and beyond twelve hours it becomes the dominant determinant of outcome.

The reperfusion component follows a different and less intuitive curve, and this is the part of the question that the clinical literature cannot answer directly. Reperfusion injury requires tissue that is still metabolically capable of generating an oxidative burst, of recruiting neutrophils and of executing regulated cell death. A testis rendered frankly necrotic by prolonged anoxia cannot mount that response, so the injury attributable specifically to the restoration of flow should not be expected to increase monotonically with ischemic time. On mechanistic grounds it should be small when ischemia is brief, because little substrate for injury has accumulated, maximal over an intermediate interval, and progressively displaced by irreversible necrosis as ischemia lengthens. We advance this as an inference from the biology rather than as a measured result, because the experiment that would establish the shape of the curve directly has not been performed in any species. What the experimental literature does establish is that the injury is duration-dependent and that its threshold is low. Graded torsion in the prepubertal rat produced histological damage that scaled with the duration of cord torsion [39], comparable grading has been shown for the degree of torsion and subsequent fertility [40] and for testicular steroidogenesis [41], and a single hour of 720 degree torsion in the rat is already sufficient to produce germ cell specific apoptosis, the first labeling appearing four hours after repair together with leukocyte margination, diapedesis and a measurable rise in lipid peroxidation products [16]. Two hours of torsion in the mouse disrupts the seminiferous epithelium and reduces both testis weight and daily sperm production [18]. The threshold for a reperfusion-mediated injury in rodents therefore lies at or below one hour, which is very much shorter than the interval at which organ survival becomes doubtful in patients.

Three consequences follow for the clinical question the reader is likely to be asking. The first is that reperfusion injury is not a late phenomenon confined to the boys who present after long delays. If one hour of ischemia suffices in a rodent, then essentially every patient who reaches theater with a twisted cord sustains some reperfusion component, including those explored within the six hour window whose testes are anatomically salvaged and who are conventionally regarded as good outcomes. That is consistent with the functional data in Section 2.3, where deficits are demonstrable in men whose surgery was timely and technically successful. The second is that the population in which a pharmacological adjunct has the largest expected effect is probably the intermediate group, meaning boys presenting between roughly six and twenty-four hours, because that is where a marginally viable organ is being reperfused and where the outcome is genuinely undetermined. Below six hours most testes survive anatomically and the residual deficit, although real, is small, so the effect size available to a drug is correspondingly small. Beyond twenty-four hours much of the parenchyma is already lost and the reperfusion component becomes a minor part of a larger irreversible injury. The third is that symptom duration is therefore the correct stratification variable for a trial and that its strata must be defined prospectively rather than by post hoc cut points, a point we return to in Section 9.3. It should be said plainly that the interval over which the human reperfusion injury unfolds has never been measured, and characterizing it is one of the specific gaps we identify in Section 11 as needing observational work before a trial is designed.

### 2.6. How Long the Salvaged Testis Should Be Monitored

A question that follows directly from the preceding subsections, and one that general clinicians ask more often than the experimental literature acknowledges, is how long a boy whose testis has been salvaged should be followed. The evidence supports a reasonably specific answer for organ volume and a much less specific one for function. Atrophy after successful detorsion and orchiopexy is common and it declares itself early in most cases, although reported frequencies vary widely between series [42,43]. In a retrospective cohort followed for a median of twelve and a half months, atrophy developed in twenty of thirty-seven salvaged testes, the median interval to detection was also twelve and a half months with a range of two to eighty-eight months, and all but two boys were clinically atrophic by fourteen months, the two exceptions being identified at thirty-five and eighty-eight months after they had defaulted from follow-up [42]. Prolonged pain, a black or hemorrhagic intraoperative appearance and preoperative parenchymal heterogeneity are the factors most consistently associated with later volume loss on ultrasound performed three to six months after surgery [44], and sonographic reassessment at two to three months has been used for the same purpose after successful manual detorsion [45]. Long-term ultrasound evaluation of boys operated for torsion confirms that structural abnormality of the salvaged organ persists well beyond the first year [46].

Volume is not function, and here the monitoring question becomes harder. Endocrine and exocrine recovery follow their own timetable. Serial measurement after surgery for torsion showed inhibin B reaching its nadir in the third postoperative week and returning to the reference range by the twelfth, while antisperm antibody titres in the same patients remained elevated for at least twenty-six weeks [47]. The historical prospective series reviewed patients at three to six months with hormone profiles, semen analysis and antibody studies [48,49], which is early enough to capture atrophy but far too early to characterize spermatogenesis in a boy who has not completed puberty. That is the central practical difficulty and it has no procedural solution. The endpoint that matters most cannot be measured until the patient is postpubertal, so in a boy operated at twelve or thirteen there is a gap of several years between the injury and the first informative semen analysis. Studies that do reach that point confirm the deficit is still detectable, with reduced testicular volume, altered gonadotropin and inhibin B concentrations and elevated seminal oxidative stress in adults with a history of torsion [9,10,26].

On the balance of this evidence we would suggest, as a pragmatic scheme rather than as a guideline recommendation, that a boy with an anatomically salvaged testis is assessed clinically and by ultrasound at approximately three months, that a second assessment at twelve months is offered because a meaningful proportion of atrophy is detected between those two points, and that families are told explicitly at discharge that late atrophy occurs and that a semen analysis after the completion of puberty is worth obtaining. The evidence behind the specific intervals is observational and the published cohorts do not agree closely on how much atrophy appears after the first three months, so we present this as a reasonable default rather than as a validated protocol. For the trial we propose in Section 9 the same logic applies in reverse. A feasibility study cannot wait for a fertility endpoint, so it has to be built around a volumetric and biomarker endpoint at three to six months together with a prespecified plan for tracing the cohort into adulthood.

## 3. Redox and Inflammatory Pathophysiology of Testicular Ischemia–Reperfusion Injury

**Figure 1:**
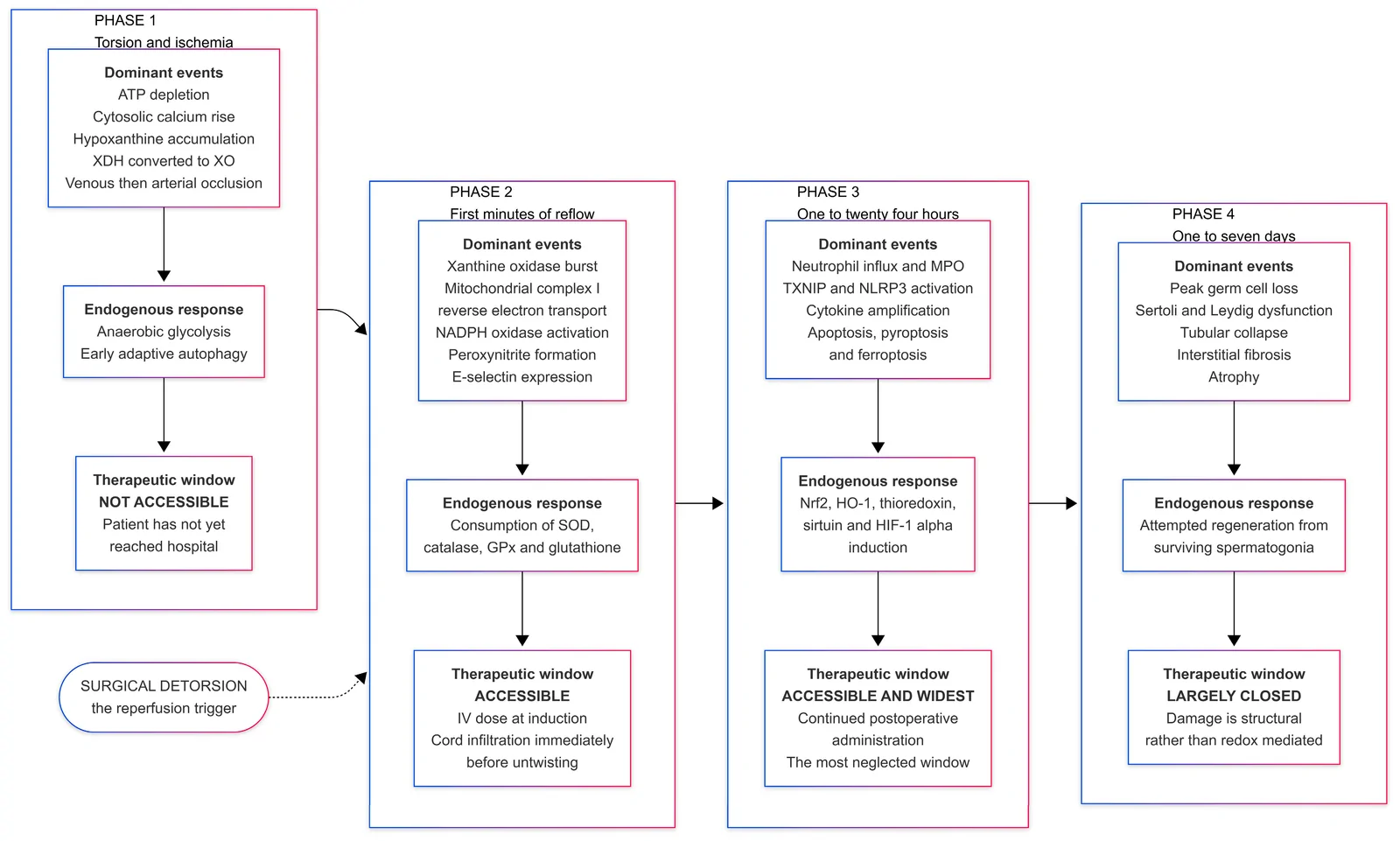
Temporal architecture of testicular ischemia–reperfusion injury and the corresponding windows for pharmacological intervention. The therapeutic opportunity that the experimental literature has most consistently exploited lies before the first column and does not exist in clinical practice. The opportunity that clinical practice actually offers lies in the third and fourth columns and is the least studied.

A drug can only be evaluated sensibly against a clear account of the process it is meant to interrupt. This section therefore sets out the mechanism in the detail required for the pharmacological appraisal that follows, with particular attention to the temporal sequencing of events, because timing turns out to be the single most consequential and the single most neglected variable in the experimental literature. Figure 1 sets out the temporal architecture of the injury and the intervention windows that follow from it, and Table 1 lists the individual sources of reactive species alongside the agents directed at each of them.

### 3.1. The Ischemic Phase

Torsion of the cord occludes the low-pressure venous return before it occludes arterial inflow, which is why the earliest change is congestion and edema rather than pallor. As intratesticular pressure rises, arterial perfusion fails and the tissue becomes anoxic. Oxidative phosphorylation ceases, adenosine triphosphate falls, and the cell reverts to anaerobic glycolysis with the accumulation of lactate and protons. Adenosine triphosphate-dependent ion pumps fail, sodium and calcium enter the cytosol, and the mitochondrial permeability transition pore is progressively primed. In parallel, adenine nucleotides are catabolized to hypoxanthine, and the constitutive xanthine dehydrogenase is converted by calcium-activated proteolysis and by thiol oxidation into xanthine oxidase, an enzyme that will use molecular oxygen as an electron acceptor the moment oxygen becomes available [11,12,50].

Two features of this phase deserve emphasis. First, ischemia alone, if sufficiently prolonged, is lethal to the tissue regardless of what follows, which is why very late presentations are unsalvageable and why no pharmacological strategy can be expected to help them. Second, ischemia of intermediate duration does comparatively little irreversible damage on its own. Classical work in the rat showed that testicular function can be substantially rescued if perfusion is restored within a defined window, and that the histological devastation characteristic of torsion develops during and after the restoration of blood flow rather than during the occlusion itself [16,30]. The therapeutic target is therefore not the ischemia. It is the reperfusion.

### 3.2. The Oxidative Burst at Reperfusion

When the cord is untwisted, oxygen returns to a tissue that has been chemically prepared to misuse it. Four sources of reactive species dominate, and they operate on different timescales. Xanthine oxidase acting on accumulated hypoxanthine produces superoxide and hydrogen peroxide within seconds to minutes. Succinate accumulates during ischemia and is rapidly oxidized at complex II once oxygen returns, which drives reverse electron transport at complex I and generates superoxide during the first minutes of reflow. This mechanism was defined in the heart and the brain and has not, to our knowledge, been demonstrated directly in the testis, so its inclusion here is an extrapolation rather than an established feature of this organ. The nicotinamide adenine dinucleotide phosphate oxidase family, and in the testis particularly the NOX2 and NOX4 isoforms expressed in endothelium, peritubular cells and infiltrating leukocytes, contributes over minutes to hours [51,52]. Finally, nitric oxide synthase becomes uncoupled and inducible nitric oxide synthase is transcriptionally upregulated, so that nitric oxide reacts with superoxide to yield peroxynitrite, a species that nitrates tyrosine residues and inactivates mitochondrial aconitase and manganese superoxide dismutase [12,50].

The importance of the nicotinamide adenine dinucleotide phosphate oxidase pathway in this specific organ has been established directly. Pharmacological inhibition of the enzyme in the rat torsion model reduces germ cell apoptosis and endoplasmic reticulum stress and preserves spermatogenesis [53], and oxidative imbalance driven by the same enzyme system has been shown to activate the thioredoxin interacting protein and nucleotide-binding oligomerization domain-like receptor family pyrin domain containing 3 axis after reperfusion [54]. It is worth noting in passing that the testis also expresses NOX5 in the germ line, where controlled superoxide generation participates in normal sperm function, which is an early warning that blanket suppression of oxidant signaling in this organ is not self-evidently benign [55,56,57].

### 3.3. Why the Testis Is Unusually Vulnerable

Several properties conspire to make the testis a poor place for an oxidative burst. The organ normally functions at an oxygen tension considerably lower than most somatic tissues, so that even modest reductions in perfusion move it towards anoxia, and the countercurrent arrangement of the pampiniform plexus that serves thermoregulation offers little reserve. The germinal epithelium is exceptionally rich in polyunsaturated fatty acids, notably docosahexaenoic and docosapentaenoic acid, which are ideal substrates for the propagation of lipid peroxidation chains and whose oxidation generates cytotoxic aldehydes including malondialdehyde and four-hydroxynonenal [58,59]. Spermatogenesis involves an enormous rate of cell division and of deoxyribonucleic acid replication, so the tissue is intrinsically sensitive to genotoxic stress, and the haploid germ cell has a very limited capacity for base excision repair. Much of the antioxidant reserve of the tubule, and the ionic and nutritional environment on which meiosis depends, lies behind the Sertoli cell tight junctions, and the blood–testis barrier is itself disrupted by circulatory hypoxia [60].

Superimposed on this is an immunological peculiarity. The adluminal compartment is an immune-privileged site containing autoantigenic germ cell epitopes that were never presented during the establishment of central tolerance. Breach of the barrier by ischemia and reperfusion exposes these antigens to a testis that is simultaneously being infiltrated by activated leukocytes, and the resulting disturbance of the local immune microenvironment has been proposed as one mechanism linking torsion to persistent subfertility and to the appearance of antisperm antibodies [61]. Finally, mitochondrial function is directly and durably impaired. Torsion and detorsion in the rat reduce the activity of oxidative phosphorylation complexes in the testis, so the organ emerges from the event with a diminished bioenergetic capacity at exactly the moment when repair processes are most demanding [62].

Because this immunological route from reperfusion to subfertility is asserted more often than it is examined, it is worth setting out what the evidence actually supports. The proposed mechanistic chain has four links. Ischemia and reperfusion disrupts the Sertoli cell tight junctions that constitute the blood–testis barrier [60], autoantigenic haploid germ cell epitopes that were never subject to central tolerance reach the interstitium and the draining lymphatics, the neutrophil and macrophage infiltrate elicited by reperfusion supplies the cytokine and costimulatory environment in which those antigens can be presented rather than ignored, and a humoral response against sperm antigens follows. The first three links are well documented in the torsion literature and are described in the subsections below. The fourth is the contested one, and its most informative feature is that it appears to be strongly duration-dependent. In a rat, torsion of 720 degrees followed by detorsion produced antisperm immunoglobulin G detectable in the contralateral testis one month later, and antibody formation was found only in animals whose torsion had lasted twelve or twenty-four hours and not in those torted for shorter intervals [63]. Rabbit experiments reached a comparable conclusion for antibody production after unilateral torsion [64]. The experimental picture is therefore that autoimmunization requires an ischemic interval that is much longer than the interval needed to produce germ cell apoptosis, which—as set out in Section 2.5—is at or below one hour. Reperfusion injury and autoimmunization are not two names for the same process, and they do not share a threshold.

The human evidence is genuinely divided and we think the honest summary is that it does not establish autoimmunization as a major mechanism of subfertility after torsion. On the positive side, autoantibodies against Leydig cells were demonstrated in patients after spermatic cord torsion [65], and twenty-four men evaluated late after torsion showed elevated seminal antisperm antibody titres both in those treated by orchiectomy after a median ischemic interval of forty-eight hours and in those treated by orchiopexy after a median of seven hours, with no significant difference between the two groups [66]. Serial measurement after orchiectomy showed antisperm antibody levels rising postoperatively and remaining elevated for at least twenty-six weeks [47]. On the negative side, forty-seven men reviewed between two and ten years after torsion showed abnormalities of exocrine or endocrine gonadal function in thirty-six cases, that is in seventy-seven percent, and yet not one of them showed evidence of testicular autoimmunization on sperm agglutination, sperm immobilization or mixed agglutination testing; eleven patients followed serially for three to six months after acute torsion developed no transient sperm antibodies either [48]. A recent prospective study of twenty-eight boys operated on for torsion found no serum antibodies against sperm or Leydig cells at all, and reported instead raised follicle stimulating hormone and anti-Müllerian hormone in the prepubertal subgroup, which its authors interpreted as evidence that primary gonadal dysfunction predisposes to torsion rather than the reverse [67].

Three interpretive points follow and they bear on how much weight this mechanism should carry. The first is a problem of causal direction that the antibody literature shares with the whole outcome literature in torsion. Contralateral testicular biopsies taken at the time of orchiopexy in fifty-six prospectively studied patients showed pre-existing partial maturation arrest of spermatogenesis in twenty of thirty-five. Fifteen of the nineteen men with that abnormality were subsequently oligozoospermic, whereas every man with normal contralateral histology had a sperm concentration within the normal range. In the same cohort the duration of torsion correlated closely with the degree of atrophy and yet showed no association with subsequent sperm concentration [49]. A dysplastic testis is both more likely to twist and more likely to be subfertile, so an association between torsion and impaired semen quality, or between torsion and detectable autoantibodies, cannot be assumed to be causal. The second point is methodological. The positive and negative human studies used different assays, different specimens, serum in some and seminal plasma in others, different positivity thresholds and different intervals after surgery, in cohorts of a few dozen patients. This is the same pattern of heterogeneity that we criticize in the preclinical literature in Section 6.1, and it produces the same outcome, which is a question still open after four decades. The third point is one of proportion. Even if antisperm antibodies do form in a subset of patients after prolonged torsion, their quantitative contribution to the functional deficit has never been estimated, and no study has shown that they account for a meaningful share of it. An antibody-mediated contribution is plausible in the long ischemia group and unproven as a general mechanism.

This matters for the therapeutic argument of the review and not only for the pathophysiology. If the immunological route contributes materially, then an agent that preserves the integrity of the blood–testis barrier during reperfusion would possess a mechanism of benefit that a pure radical scavenger does not, and barrier integrity would become a rational secondary endpoint. If it does not contribute, then measuring antibodies in a first in human study would consume resources for no information. Because the evidence does not resolve the question, and because the experimental data place the threshold for autoimmunization well beyond the interval at which most patients are operated, we would place antisperm antibody measurement among the exploratory rather than the confirmatory biomarkers discussed in Section 9.4.

### 3.4. Neutrophils, Endothelium and the Amplification Loop

The initial chemical burst is large but brief. What converts it into sustained tissue destruction is the recruitment of leukocytes, and the evidence for this in the testis is as strong as in any organ. Ischemia and reperfusion of the mouse testis induces expression of endothelial adhesion molecules, notably E-selectin, on subtunical venules, followed by neutrophil rolling, firm adhesion and transmigration. Germ cell apoptosis follows this recruitment closely in space and in time, and animals rendered deficient in the relevant adhesion pathway are substantially protected, which establishes the neutrophil as a necessary rather than an incidental participant [17,18]. Activated neutrophils then supply a second and much larger wave of oxidants through their own nicotinamide adenine dinucleotide phosphate oxidase and through myeloperoxidase, which converts hydrogen peroxide into hypochlorous acid and chloramines. Tissue myeloperoxidase activity is for this reason one of the most widely used surrogate markers of injury severity in the experimental literature, and its suppression is reported by almost every agent claimed to be protective [51].

The temporal profile that emerges from this work is clinically important. Germ cell apoptosis in a rat or mouse is not maximal at the moment of reperfusion. It rises over hours and peaks approximately one day after the repair of torsion [16,17]. If that timing translates even approximately to a human, then the pharmacological window is not confined to the operating theater. It extends through the first postoperative day, which is precisely the period in which a hospitalized patient is easiest to treat and to monitor. This single observation should have driven the field towards postoperative dosing schedules many years ago. It must be said plainly, however, that the human temporal profile is unknown. No study has characterized the time course of germ cell death after detorsion in man, spermatogenic kinetics are not identical in rodents and humans, and the whole of the dosing schedule proposed in Section 9.2 rests on the assumption that the rodent timing transfers. That assumption is testable without an interventional trial, using delayed orchiectomy specimens, biopsy material obtained at exploration where this is already practiced, and serial imaging and hormonal profiling, and we regard such a characterization study as the logical first step rather than an optional refinement.

### 3.5. Inflammasome Activation and Cytokine Signaling

Reactive species do not act only as direct chemical toxins. They are also signals, and one of the most consequential signaling events after testicular reperfusion is assembly of the nucleotide-binding oligomerization domain-like receptor family pyrin domain-containing 3 inflammasome. Work in the mouse demonstrated that this complex is activated after testicular ischemia and reperfusion, that its activation correlates with organ damage and with impaired spermatogenesis, and that genetic or pharmacological interference with it is protective [68]. The same group subsequently showed that oxidant-driven inflammasome activation is a shared feature of ischemia and reperfusion in brain, heart, kidney and testis, which places the testis firmly inside the general biology of reperfusion injury rather than in a compartment of its own [69]. The upstream link between oxidant load and inflammasome assembly appears to run through thioredoxin-interacting protein, which dissociates from oxidized thioredoxin and binds the receptor, and this axis has been demonstrated specifically in the reperfused testis [54,70].

Downstream, caspase 1 matures interleukin-1β and interleukin-18, tumor necrosis factor alpha rises, cyclooxygenase two is induced and further neutrophils are recruited, closing a feed-forward loop between oxidant generation and inflammation. This is the reason that agents with no antioxidant chemistry whatsoever, such as colchicine or a selective cyclooxygenase inhibitor, can reproduce much of the protection attributed to classical scavengers, and it is an important caution against reading every positive result in this literature as evidence for a purely redox mechanism.

### 3.6. Modes of Regulated Cell Death

For most of the history of this field the injury was described as apoptosis, and apoptosis is certainly present. The canonical pathway has been mapped in the rat testis, with downregulation of B-cell lymphoma two, a rising ratio of Bcl-2-associated X protein to B-cell lymphoma two, cytochrome c release, caspase nine and caspase three activation and internucleosomal deoxyribonucleic acid fragmentation, together with activation of the stress kinases c-Jun N-terminal kinase and p38 [17,70]. It is now clear that this is an incomplete picture. Pyroptosis, the caspase 1 and gasdermin-dependent lytic death that follows inflammasome activation, has been demonstrated in the reperfused testis and shown to be regulated by non-coding ribonucleic acid networks [71]. Ferroptosis, an iron-dependent form of death driven by the peroxidation of membrane phospholipids and by failure of glutathione peroxidase 4, has been shown in Sertoli cells subjected to oxygen and glucose deprivation followed by reoxygenation [72], and pharmacological protection of the reperfused testis has been achieved specifically by restoring the nuclear factor erythroid 2-related factor 2, heme oxygenase 1 and glutathione peroxidase 4 axis [73].

Autophagy occupies an ambiguous position. During the ischemic phase it appears to be adaptive, sustaining energy supply and clearing damaged organelles, whereas during reperfusion the same signaling machinery contributes to death, and the switch between the two appears to be governed at least in part by c-Jun N-terminal kinase activity [74]. The practical implication for drug development is uncomfortable but important. Because several distinct death programs operate in parallel and are differently timed, an agent that blocks only one of them may simply redirect cells into another, and the histological endpoint will improve less than the molecular endpoint suggests. This may be one reason why effect sizes reported for biochemical markers in this literature are so much larger than the effect sizes reported for spermatogenic scores.

### 3.7. Endogenous Cytoprotective Programs

The testis is not passive. Reperfusion activates the nuclear factor erythroid 2-related factor 2 pathway, which dissociates from Kelch-like ECH-associated protein 1 and drives transcription of heme oxygenase 1, NAD(P)H quinone dehydrogenase 1, glutamate cysteine ligase and a wide battery of phase two enzymes. Agents that succeed in this model very often turn out on mechanistic dissection to be activators of this pathway rather than stoichiometric scavengers, which is theoretically attractive because a transcriptional response amplifies a small chemical stimulus into a large and sustained antioxidant capacity [75,76]. The thioredoxin system, the sirtuin family and hypoxia inducible factor one alpha form additional layers of the same adaptive response, and hypoxia inducible factor one alpha in particular has been shown to be involved in experimental testicular ischemia and reperfusion and to be modifiable pharmacologically [77,78].

There is an obvious therapeutic corollary and an equally obvious risk. If endogenous defenses can be pre-armed, then interventions given before the oxidative burst should outperform interventions given after it, and this is exactly what the experimental literature shows. It is also exactly what cannot be done in a patient who arrives in an emergency department with a testis that has already been twisted for nine hours. Section 6 returns to this point, because it is in our judgment the central methodological failure of the field.

### 3.8. Endocrine and Long-Term Structural Consequences

Attention has concentrated on the germ cell, but the Leydig cell is also affected. Experimental torsion reduces intratesticular and serum testosterone, alters the expression of steroidogenic acute regulatory protein and of the cytochrome P450 side chain cleavage enzyme, and produces a compensatory rise in luteinizing hormone [29]. Sertoli cell function is disturbed in parallel, which is reflected clinically in altered inhibin B concentrations in men with a history of torsion [9]. Over the longer term the tubules that survive show reduced diameter, thinning and disorganization of the germinal epithelium, and interstitial fibrosis, and the Johnsen score, which remains the standard semiquantitative measure of spermatogenic completeness, falls in proportion to the severity and duration of the initial insult [31,79]. It is these chronic structural outcomes, rather than any biochemical measurement made four hours after detorsion, that define whether a treatment has actually worked.

## 4. Endpoints and Biomarkers in Experimental and Clinical Work

Before any agent can be judged, the measuring instruments have to be examined, because much of the apparent consistency of this literature is an artifact of the endpoints chosen. Four families of measurement are in use, and they differ enormously in how closely they track the outcome a patient would care about. Table 2 arranges them in order of that proximity.

The first family is tissue redox chemistry. Malondialdehyde as a marker of lipid peroxidation, superoxide dismutase, catalase and glutathione peroxidase activities, reduced glutathione concentration, total antioxidant status and total oxidant status, and myeloperoxidase activity as a proxy for neutrophil burden are reported in the overwhelming majority of published studies [11,15,80]. They are cheap, they are reproducible within a laboratory, and they respond briskly to almost any intervention with reducing chemistry. That last property is precisely the problem. A compound that lowers tissue malondialdehyde four hours after detorsion has demonstrated that it is a reducing agent that reaches the testis. It has not demonstrated that it preserves spermatogenesis.

The second family is molecular and histological. Terminal deoxynucleotidyl transferase-mediated deoxyuridine triphosphate nick end labeling, caspase 3 immunoreactivity, the ratio of Bcl-2-associated X protein to B-cell lymphoma two, inflammasome and cytokine expression, seminiferous tubule diameter, germinal epithelial height and the Johnsen score belong here [16,17,79]. These are closer to the biology that matters, and the Johnsen score in particular has the advantage of being the same instrument used on human testicular biopsies, which makes it one of the few genuinely translational readouts available. Its weakness is observer dependence, and in our reading of the studies summarized below blinded scoring by more than one pathologist remains the exception rather than the rule.

The third family is functional and gametic. Epididymal sperm concentration, motility and morphology, sperm deoxyribonucleic acid fragmentation, chromatin condensation and the oxidized eight-hydroxy-two-deoxyguanosine adduct load, and intratesticular and serum testosterone belong here [81,82]. These endpoints require the animal to be kept alive for at least one full spermatogenic cycle, which in the rat means about eight weeks, and they are correspondingly rare. The fourth family, actual fertility measured by mating and litter size, is rarer still, and its near absence from the field is remarkable given that fertility is the reason anyone is interested in the problem at all.

In humans, the corresponding instruments are semen analysis according to current World Health Organization criteria, sperm deoxyribonucleic acid fragmentation, seminal oxidation reduction potential, ultrasonographic testicular volume, and the hormonal profile of follicle stimulating hormone, luteinizing hormone, testosterone and inhibin B. Long-term follow-up work in men treated for torsion has shown all of these to be measurable and to differ from controls, which establishes that a clinical trial in this field would not lack outcome measures [9,10,26]. The obstacle has never been the absence of endpoints. It has been the absence of trials.

## 5. The Antioxidant Armamentarium

What follows is an appraisal organized by mechanism rather than by botanical or pharmacological family, because the mechanistic grouping is what determines whether two positive results are mutually corroborating or merely repetitive. Throughout, we record not only the direction of effect but the variable that the field has most consistently ignored, which is when the drug was given relative to the reperfusion event. Because that variable is central to the argument of this review, we define it more finely than is usual. Pretreatment means administration beginning before torsion was induced, in some studies for days or weeks. Intraischemic means administration during the ischemic interval, and this deserves a category of its own, because such an agent is present in the circulation before the oxidative burst and yet cannot reach an organ whose perfusion is absent, so that it in fact arrives in the testis at the moment of reflow. At detorsion means administration timed to the restoration of flow. Post-detorsion means administration begun after flow had already been restored. Only the last two categories correspond to anything that a clinician can do, and the older undifferentiated term peritorsional is not used here because it conflates the second category with the third. An agent is included where at least one primary experimental study in a torsion and detorsion or testicular ischemia and reperfusion model was identified, and Table 3, Table 4 and Table 5 summarize these agents together with the species studied, the effects reported and, where the primary reports state it, the timing of administration. We draw attention to the fact that timing was extractable for only a minority of the entries, and that it is absent for almost all of the plant-derived compounds in Table 4. That is not a defect of the tables. It is one of the findings of this review, and Section 6.2 quantifies it.

### 5.1. Direct Scavengers, Vitamins and Carotenoids

The earliest and conceptually simplest strategy is to supply a molecule that reacts with the offending species stoichiometrically. Raxofelast, a hydrophilic analog of vitamin E, reduced testicular ischemia and reperfusion injury in the rat and, importantly, was one of the first agents whose mechanism was pursued as far as the mitogen-activated protein kinase cascades that lipid peroxidation activates [83,84]. Lycopene, the carotenoid of the tomato, attenuated testicular injury in two independent laboratories using different torsion protocols, which is the kind of cross-laboratory replication the field otherwise almost entirely lacks [85,86]. Astaxanthin, a xanthophyll carotenoid with unusually favorable membrane-spanning geometry, has been examined in detail in prepubertal rats, with morphometric analysis of histological sections and with immunohistochemical and biochemical quantification of caspase 3-positive cells, malondialdehyde, superoxide dismutase and glutathione peroxidase [87,88]. That work is from our own group, and we single it out for a methodological reason rather than a chemical one. It compared administration at the moment of detorsion with administration forty-five minutes later, and found the delayed schedule to be at least as effective, which is one of the very few direct experimental tests of the timing question that this review identifies as central. Because the work is our own, and because it has not been replicated independently, we place no more weight on it than a single laboratory finding can bear. Independent confirmation that a delayed schedule is not inferior to an immediate one would be among the most useful single experiments the field could now perform.

Ascorbate has generally been studied in combination rather than alone, for example together with dopamine in a design intended to address both the oxidative and the vasomotor components of the injury [89]. The overall picture for this class is of consistent benefit on biochemical markers, more modest benefit on histology, and almost no data on gametic function. The pharmacological objection to pure scavengers is also worth stating early. A stoichiometric antioxidant must be present in molar quantities comparable to the oxidant burden at the site of generation, which is a demanding requirement for a burst that occurs inside mitochondria and within neutrophil phagosomes over a period of minutes. We return to ascorbate in Section 7.4, where we consider whether its favorable safety profile is by itself an argument for empirical clinical use.

### 5.2. Thiol Donors and Support of the Glutathione System

A more sophisticated approach is to reinforce the endogenous glutathione system rather than to substitute for it. N-acetylcysteine, which is already licensed, already used in children, and already familiar to every emergency department, reduced malondialdehyde and raised glutathione peroxidase in the rat torsion model and improved histological outcome [90]. Alpha lipoic acid, which is unusual in being active in both oxidized and reduced forms and in both aqueous and lipid compartments, was similarly protective [91]. Taurine, which is not a classical antioxidant but stabilizes membranes and scavenges hypochlorous acid generated by myeloperoxidase, has shown benefit alone and in combination with carnosine [92,93]. Thymoquinone, tested head-to-head against a combination of conventional antioxidants, was at least comparable [94].

This class deserves particular attention in any translational plan. N-acetylcysteine in particular satisfies almost every practical criterion that an emergency adjunct must meet. It has an intravenous formulation, a well-characterized pediatric dosing schedule derived from decades of use in paracetamol poisoning, an excellent safety record, negligible cost, and a mechanism that is upstream rather than stoichiometric. If a single molecule had to be selected today for first-in-human evaluation on pragmatic grounds, it would be difficult to argue against it. Two caveats belong alongside that statement. The experimental evidence for N-acetylcysteine in this particular model rests on very few reports, so the case for it is pragmatic rather than evidential and preclinical replication should precede any human study. And an excellent safety record is not the same thing as an absence of risk, a point taken up in Section 7.3.

### 5.3. Indoleamines, Steroids and Peptide Hormones

Melatonin is, by a wide margin, the most studied agent in this field, and the volume of work devoted to it illustrates both the strengths and the weaknesses of the literature. It reduces proinflammatory cytokine expression after testicular reperfusion [95], improves spermatogenesis and reduces atrophy in unilateral torsion and detorsion models [96,97], preserves testicular histology and lowers oxidative markers [98,99], protects the contralateral as well as the ipsilateral organ in at least one careful comparison [100], and has been evaluated against ozone therapy [101], against colchicine [102] and in combination with metformin [103]. Several dozen further studies could be added. Melatonin has an outstanding safety profile, crosses every biological membrane readily, acts both as a direct scavenger and as an inducer of antioxidant enzyme transcription, and is already given to children for sleep disorders.

And yet the same body of work exposes the central problem. The doses used vary across more than three orders of magnitude, from micrograms to tens of milligrams per kilogram, the timing varies from three weeks of pretreatment to a single injection after detorsion, the comparator groups differ, and the results are almost uniformly positive regardless of any of these choices. A literature in which every dose and every schedule works equally well is not a literature that can guide a human dose selection. Among other hormonal agents, dehydroepiandrosterone reduced apoptosis [104], erythropoietin was protective in two independent studies including a direct comparison with sildenafil [105,106], urocortin acted as an anti-apoptotic protein in a reperfused rat testis [107], and melanocortin four receptor activation protected the testis by triggering the cholinergic anti-inflammatory pathway, which is mechanistically distinct from anything else in this section and remains underexplored [108].

**Table 3:** Direct scavengers, thiol donors and hormonal agents evaluated in experimental testicular ischemia–reperfusion injury.

Agent	Class and Principal Mechanism	Model and Timing of Administration	Main Reported Effects	References
Raxofelast	Hydrophilic vitamin E analog, chain-breaking scavenger	Rat, intraischemic	Reduced lipid peroxidation and MAPK activation, improved histology	[83,84]
Lycopene	Carotenoid, singlet oxygen quencher	Rat, pretreatment and around torsion and detorsion	Lower MDA, preserved tubular architecture	[85,86]
Astaxanthin	Xanthophyll carotenoid spanning the lipid bilayer	Prepubertal rat, at detorsion and 45 min after detorsion	Fewer caspase 3-positive cells, lower MDA, higher SOD and GPx	[87,88]
Ascorbate with dopamine	Aqueous scavenger combined with vasomotor support	Rat, timing reported only as around torsion and detorsion	Improved biochemical and histological indices	[89]
N-acetylcysteine	Cysteine donor, replenishes glutathione	Rat, timing reported only as around torsion and detorsion	Lower MDA, higher GPx, improved histology	[90]
Alpha lipoic acid	Amphipathic redox couple, regenerates other antioxidants	Rat, pretreatment	Reduced oxidative injury and apoptosis	[91]
Taurine and carnosine	Membrane stabilization, scavenging of hypochlorous acid	Rat, pretreatment and around torsion and detorsion	Reduced peroxidation, preserved spermatogenesis	[92,93]
Thymoquinone	Quinone from Nigella sativa, Nrf2 activation	Rat, compared with combined antioxidants	Comparable or superior to conventional combination	[94]
Melatonin	Indoleamine, direct scavenger and transcriptional inducer	Rat, every schedule from 3 weeks pretreatment to single post-detorsion dose	Lower cytokines and MDA, improved spermatogenesis, reduced atrophy	[95,96,97,98,99,100,101,102,103]
Dehydroepiandrosterone	Adrenal steroid, anti-apoptotic	Rat, timing reported only as around torsion and detorsion	Reduced germ cell apoptosis	[104]
Erythropoietin	Cytokine, anti-apoptotic and anti-inflammatory	Rat, timing reported only as around torsion and detorsion, one head-to-head with sildenafil	Reduced injury scores and apoptosis	[105,106]
Urocortin	Corticotropin-releasing factor family peptide	Rat, timing reported only as around torsion and detorsion	Anti-apoptotic protection of germinal epithelium	[107]
Melanocortin 4 receptor agonist	Cholinergic anti-inflammatory pathway activation	Rat, timing reported only as around torsion and detorsion	Reduced inflammation and tissue injury	[108]

### 5.4. Polyphenols and Other Plant-Derived Compounds

This is numerically the largest group and analytically the least satisfying. Flavonoids including quercetin, apigenin and hesperidin have each been shown to reduce oxidative markers and preserve histology after torsion and detorsion in the rat [109,110,111]. The green tea catechin epigallocatechin gallate is protective, and in one study its effect was traced specifically to modulation of germ cell apoptosis through a survivor activating factor enhancement and nuclear factor erythroid 2-related factor 2-dependent route rather than to direct scavenging, which is a considerably more interesting claim than the biochemistry alone would support [112,113]. The isoflavone genistein alleviated spermatogenic damage by suppressing abnormal matrix metalloproteinase signaling [114]. Salvianolic acid B, ligustrazine, proanthocyanidin, grape seed extract, Korean red ginseng, goji berry extract and ternatin have all been reported as protective [115,116,117,118,119,120,121], as have salidroside acting through the nuclear factor erythroid 2-related factor 2, heme oxygenase 1 and glutathione peroxidase 4 axis to inhibit ferroptosis [73] and sulforaphane acting as a canonical inducer of the same transcriptional program [75].

Read individually, these are competent studies. Read together, they raise an uncomfortable question. Dozens of structurally unrelated plant extracts, tested in essentially the same model with essentially the same readouts, all produce essentially the same result. Either the testis is protected by almost any reducing substance that reaches it, in which case the specific chemistry is of little interest and the effect is unlikely to be large, or the uniformity of the findings reflects publication practice rather than pharmacology. Both explanations are probably partly correct. The subgroup that deserves continued attention is the one in which protection was traced to a defined transcriptional or cell death pathway and confirmed by pathway manipulation rather than by correlation, because those studies make a falsifiable claim. Bioavailability is the other filter that should be applied ruthlessly. Curcumin, resveratrol and most dietary flavonoids have oral bioavailability so poor that the plasma concentrations achieved in humans are orders of magnitude below those used in vitro, and an intervention that must be given intravenously within minutes of a surgical event cannot rely on a compound with no parenteral formulation.

### 5.5. Agents Directed at Specific Oxidant Generating Enzymes

A mechanistically stronger strategy is to inhibit the enzymes that make the oxidants rather than to mop up the product. Apocynin, an inhibitor of nicotinamide adenine dinucleotide phosphate oxidase assembly, protected the reperfused rat testis [122], and direct inhibition of the same enzyme system reduced germ cell apoptosis and endoplasmic reticulum stress and preserved spermatogenesis [53]. Xanthine oxidase inhibition with allopurinol is the oldest idea in the whole field of reperfusion pharmacology and has been examined repeatedly in testicular models, generally with biochemical benefit that exceeds its histological benefit. The more potent non-purine inhibitor febuxostat has been proposed as a rationally superior successor for the ischemic phase—specifically, on the grounds that it acts before the burst rather than after it [11]. Pyrrolidine dithiocarbamate, which inhibits nuclear factor kappa B activation, and the tyrosine kinase inhibitor tyrphostin AG 556 have also been tested with positive results [123,124], and inhibition of c-Jun N-terminal kinase with SP600125 restored autophagic and apoptotic balance and reversed spermatogenic arrest [74].

The theoretical appeal of this group is that a catalytic inhibitor does not need to be present in molar excess of the oxidant, only in sufficient concentration to occupy the enzyme, which is a far more realistic pharmacological demand. The practical difficulty is specificity. Apocynin is a prodrug requiring peroxidase activation and has non-specific effects at the concentrations commonly used; blanket inhibition of nicotinamide adenine dinucleotide phosphate oxidase, nuclear factor kappa B or the stress kinases interferes with the signaling pathways that the tissue also needs for repair. The considerable body of evidence that physiological concentrations of reactive oxygen species are required for spermatogonial stem cell self-renewal is a direct warning on this point [56,57].

**Table 4:** Plant-derived compounds and enzyme directed agents evaluated in experimental testicular ischemia–reperfusion injury.

Agent	Principal Proposed Mechanism	Model and Timing Where Reported	Main Reported Effects	References
Quercetin	Flavonol, scavenging and anti-inflammatory	Rat torsion and detorsion	Reduced oxidative markers, preserved histology	[109]
Apigenin	Flavone, anti-inflammatory	Rat	Reduced tissue injury scores	[110]
Hesperidin	Citrus flavanone glycoside	Rat	Lower MDA, improved histology	[111]
Epigallocatechin gallate	Catechin, SAFE and Nrf2-dependent modulation of germ cell apoptosis	Rat	Reduced apoptosis, pathway confirmed by manipulation	[112,113]
Genistein	Isoflavone, suppression of aberrant MMP signaling	Rat	Reduced spermatogenic damage and oxidative stress	[114]
Salvianolic acid B	Polyphenol from Salvia miltiorrhiza	Rat	Attenuated injury and oxidative markers	[115]
Ligustrazine	Alkaloid from Ligusticum, anti-apoptotic	Rat	Reduced oxidative stress and apoptosis	[116]
Proanthocyanidin	Condensed tannin	Rat	Reduced ischemia–reperfusion injury	[117]
Grape seed extract	Polyphenol mixture, eNOS modulation	Rat	Lower MDA and advanced oxidation protein products	[118]
Korean red ginseng	Ginsenoside mixture	Rat	Reduced tissue injury after torsion and detorsion	[119]
Goji berry extract	Polysaccharide and carotenoid mixture	Rat	Protective against reperfusion injury	[120]
Ternatin	Flavonoid from Egletes viscosa	Rat, pretreatment	Attenuated testicular injury	[121]
Salidroside	Nrf2, HO-1 and GPX4 axis, ferroptosis inhibition	Rat and TM4 Sertoli cells	Pathway confirmed by Nrf2 transfection rescue	[73]
Sulforaphane	Isothiocyanate, canonical Nrf2 inducer	Rat	Reduced injury after torsion and detorsion	[75]
Apocynin	NADPH oxidase assembly inhibitor	Rat	Reduced oxidative injury	[122]
NADPH oxidase inhibition	Direct suppression of the principal ROS source	Rat	Less germ cell apoptosis and ER stress, preserved spermatogenesis	[53]
Allopurinol and febuxostat	Xanthine oxidase inhibition during the ischemic phase	Rat, proposed for the ischemic window	Biochemical benefit exceeding histological benefit	[11]
Pyrrolidine dithiocarbamate	NF-kappa B inhibition	Rat	Reduced inflammatory injury	[124]
Tyrphostin AG 556	Tyrosine kinase inhibition	Rat	Reduced ischemia–reperfusion injury	[123]
SP600125	c-Jun N-terminal kinase inhibition	Rat	Restored autophagy and apoptosis balance, reversed spermatogenic arrest	[74]

### 5.6. Repurposed Clinical Drugs

A distinct and, for translational purposes, far more promising group consists of compounds that are already licensed for human use. Simvastatin attenuated testicular injury after torsion and detorsion, and the same laboratory had earlier shown that induction of heme oxygenase 1 is protective in this model, which supplies a coherent mechanism [76,125]. Phosphodiesterase five inhibitors have been examined repeatedly, including vardenafil in a porcine model that is anatomically closer to the human than the rat, and udenafil in comparison with piracetam and dexmedetomidine [126,127]. The alpha two adrenoceptor agonist dexmedetomidine, the intravenous anesthetics propofol and thiopental, and the calcium channel blocker amlodipine have all shown protection, which is of particular interest because all of them are drugs that a child undergoing emergency scrotal exploration might plausibly receive anyway as part of the anesthetic [128,129,130]. Vasoactive agents including papaverine, lidocaine and verapamil improved sperm quality after torsion and detorsion [131], and the beta blocker carvedilol, which has intrinsic antioxidant activity, was likewise protective [132].

Metformin, acting through adenosine monophosphate-activated protein kinase and the nuclear factor erythroid 2-related factor 2 pathway, reduced injury in the rat [133]. Anti-inflammatory approaches have included ibuprofen [134] and the sphingosine-1-phosphate receptor modulator fingolimod, which inhibited germ cell apoptosis after torsion and detorsion [135]. Osmotic and supportive interventions such as mannitol and dexpanthenol have also been reported as beneficial [41,136]. The strategic point is straightforward. An agent that already has a pediatric license, a parenteral formulation and an established safety profile can enter a clinical trial in this indication in a fraction of the time and at a fraction of the cost of a novel chemical entity, and this is where the field should be concentrating its attention.

### 5.7. Gasotransmitters, Oxygen-Based and Physical Strategies

Molecular hydrogen has attracted interest because it reacts preferentially with the hydroxyl radical and peroxynitrite while leaving physiologically useful species such as superoxide and hydrogen peroxide relatively untouched, which is a degree of selectivity that no conventional antioxidant offers. Both hydrogen rich saline and inhaled hydrogen gas protected the rat testis from ischemia and reperfusion injury [137,138]. Hyperbaric oxygen, which is counterintuitive as an antioxidant strategy, reduced oxidative stress, suppressed inflammation and lowered nitric oxide formation in the reperfused rat testis, presumably by preconditioning endogenous defenses rather than by any direct scavenging [139]. Ozone therapy has been compared directly with melatonin [101].

Conditioning protocols form a conceptually separate group that requires no drug at all. Ischemic preconditioning, postconditioning and remote conditioning apply brief cycles of occlusion and reflow to modify the tissue response to the definitive reperfusion, and ischemic postconditioning in particular is attractive here because it is applied after the ischemic period and therefore respects the clinical constraint that the surgeon meets the patient only after the injury has begun [20]. In the testis this would amount to a defined sequence of partial retorsion and detorsion at the end of the operation; its attraction is that it costs nothing, that it would add only a few minutes to the procedure and that it involves no medicinal product. Three qualifications are needed, however, before it is presented as ready for a human study. The protocol is entirely unspecified, since the number of cycles, the duration of each occlusion and reflow phase and the degree of retorsion have never been defined for this organ. Deliberately re-occluding the vascular pedicle of a marginally viable testis is not a risk-free maneuver and would require an explicit safety justification. And the absence of a medicinal product removes the need for a product license but not the need for ethical approval and, in many jurisdictions, for formal oversight of an interventional surgical study. What is genuinely striking is not that the intervention has not been tested in humans, but that its protocol has not yet been defined in an animal model with a spermatogenic endpoint, which is where this work should begin.

### 5.8. Cell and Vesicle-Based Approaches

Human umbilical cord multipotent mesenchymal stromal cells reduced inflammation and oxidative stress and alleviated acute injury to spermatogenic cells in an experimental model [140], and exosomes derived from adipose tissue mesenchymal stromal cells prevented torsion injury through phosphoinositide 3-kinase and mitogen activated protein kinase signaling [141]. These approaches are mechanistically interesting because a vesicle delivers a complex cargo of proteins and regulatory ribonucleic acids rather than a single chemical species, which suits a pathology with several parallel death programs. They are also, for the foreseeable future, entirely unsuited to an emergency surgical pathway in which the intervention must be available at three o’clock in the morning in a district hospital. We include them for completeness and because they may eventually inform the design of simpler mimetics, not because we regard them as near term clinical candidates.

**Table 5:** Repurposed clinical drugs, physical strategies and cell-based approaches evaluated in experimental testicular ischemia–reperfusion injury.

Intervention	Existing Clinical Status	Model and Timing	Main Reported Effects	References
Simvastatin	Licensed, oral only	Rat, timing reported only as around torsion and detorsion	Reduced injury, heme oxygenase 1-dependent	[76,125]
Vardenafil	Licensed, oral	Pig, timing reported only as around torsion and detorsion	Reduced torsion and detorsion damage in a large animal	[126]
Udenafil with piracetam and dexmedetomidine	Licensed agents	Rat	Comparative protection across three agents	[127]
Dexmedetomidine	Licensed, intravenous, used in pediatric anesthesia	Rat	Reduced torsion and detorsion damage	[128]
Propofol and thiopental	Licensed, intravenous induction agents	Rat	Prevention of ischemia–reperfusion injury	[129]
Amlodipine	Licensed, oral	Rat	Reduced injury, vasomotor and calcium-mediated mechanisms	[130]
Papaverine, lidocaine, verapamil	Licensed, injectable	Rat	Improved sperm quality after torsion and detorsion	[131]
Carvedilol	Licensed, oral, intrinsic antioxidant activity	Rat	Reduced oxidative injury	[132]
Metformin	Licensed, oral	Rat	AMPK- and Nrf2-mediated protection	[133]
Ibuprofen	Licensed, oral and intravenous	Rat	Reduced inflammatory component of injury	[134]
Fingolimod	Licensed, oral	Rat	Inhibited germ cell apoptosis	[135]
Mannitol	Licensed, intravenous	Rat	Protective in experimental torsion	[41]
Dexpanthenol	Licensed	Rat, outcome at 60 days	Reduced late testicular atrophy	[136]
Hydrogen rich saline	Investigational	Rat	Selective hydroxyl radical scavenging, reduced injury	[137]
Inhaled hydrogen gas	Investigational	Rat, given at reperfusion	Prevention of ischemia–reperfusion injury	[138]
Hyperbaric oxygen	Licensed procedure	Rat	Reduced oxidative stress, inflammation and nitric oxide	[139]
Ozone therapy	Unlicensed procedure	Rat, compared with melatonin	Comparable protection to melatonin	[101]
Ischemic postconditioning	Procedural, no medicinal product	Experimental, applied after ischemia	Protective, uniquely compatible with the clinical time course	[20]
Umbilical cord mesenchymal stromal cells	Investigational	Rat	Reduced inflammation and oxidative stress	[140]
Adipose mesenchymal stromal cell exosomes	Investigational	Rat	PI3K, AKT and MAPK-dependent protection	[141]

## 6. Reading the Preclinical Evidence Critically

Section 5 described a literature in which almost everything works. This observation should be treated as a finding about the literature rather than as a finding about biology, and this section examines why. Table 6 collects the recurring methodological features and what each of them costs in translational terms.

### 6.1. Model Heterogeneity

There is no standard model of testicular torsion. Across the studies summarized above, the species is usually a rat but is sometimes a mouse, a rabbit or a pig; the animals are sometimes prepubertal and sometimes adult; the torsion angle in the studies tabulated here ranges from 540 to 1080 degrees; the ischemic interval ranges from thirty minutes to eight hours; and the reperfusion interval before sacrifice ranges from one hour to sixty days. Each of these choices changes the biology substantially. A ninety-minute torsion at 720 degrees in an adult rat followed by four hours of reperfusion is a different disease from a four-hour torsion at 1080 degrees in a prepubertal rat followed by eight weeks of recovery, and the two cannot be pooled. The consequence is that the apparent replication of positive findings across dozens of agents is largely illusory, because almost no two studies are close enough in design for one to corroborate another. The very few instances of genuine cross-laboratory replication with a shared protocol stand out precisely because they are rare [85,86].

The relationship between the experimental model and the clinical situation is looser still. The rat testis is small, the surgical trauma of exteriorization is proportionally large, the animals are young, healthy, genetically similar, unmedicated and free of the comorbidity, delay and physiological stress that characterize a real patient, and the torsion is applied under anesthesia to a testis that was normal a moment earlier. None of this invalidates the models, which have taught the field almost everything it knows about the mechanism. It does mean that quantitative effect sizes derived from them should be treated as upper bounds rather than as predictions.

One species difference deserves particular mention, because it is rarely acknowledged and because it cuts directly against the modeling strategy that the field has adopted. Rats and mice lack a functional NOX5 gene, so the calcium regulated oxidase that is expressed in the human germ line and participates in normal sperm function does not exist in the animals in which almost all of this work has been performed, and species such as the rabbit, which retain the gene, would be required in order to study it [142]. Whatever contribution NOX5 makes to redox signaling in the reperfused human testis, and whatever risk attends its suppression, no rodent torsion model can address the question at all.

### 6.2. The Pretreatment Problem

This is in our view the single most serious defect in the field, and it is remarkable how rarely it is stated plainly. It is also, unfortunately, difficult to quantify, and that difficulty is itself informative. Of the fifty-three interventions tabulated in Table 3, Table 4 and Table 5, the timing of administration relative to torsion and detorsion could be established from the primary reports for only eighteen. Of those eighteen, five involved administration beginning before torsion was induced, in some cases for days or weeks beforehand, two were given during the ischemic interval, eight were described only in terms that place them somewhere around torsion and detorsion without further precision, and just three were unambiguously administered at or after the restoration of blood flow. In other words, for roughly two thirds of the agents in this literature the single variable that determines whether a positive result carries any clinical meaning cannot be recovered from the publication at all, and among the reports where it can be recovered the clinically relevant schedule is the rarest of the four. Three weeks of intraperitoneal melatonin or a loading regimen of alpha lipoic acid given to an animal that is subsequently torted answers a question about prophylaxis. It does not answer the question the clinician faces, which is what to give to a boy whose testis has already been twisted for nine hours and who is now on the operating table.

The distinction is not pedantic, because the mechanisms that pretreatment exploits are precisely the ones that cannot be exploited afterwards. Transcriptional induction of the nuclear factor erythroid 2-related factor 2 battery, repletion of the glutathione pool, and accumulation of a lipophilic scavenger in testicular membranes all require hours to days. An agent whose entire benefit depends on being present in the tissue before the oxidative burst begins is not a candidate for clinical use in torsion at all, however impressive its preclinical data. The intraischemic category needs further discussion, because it cuts in a direction that is not usually noticed. An agent injected during the ischemic interval in a rodent model cannot reach a testis whose perfusion has been abolished, and it therefore arrives in the organ with the returning blood at the moment of reflow. Such a study may be a closer model of clinical dosing than its own description suggests, provided that the agent acts stoichiometrically or catalytically rather than by transcriptional pre-arming. Where the reported benefit depends on induction of the nuclear factor erythroid 2-related factor 2 battery, on the other hand, the systemic exposure achieved before detorsion may have done the work, and the two possibilities cannot be separated without the pharmacokinetic data that these studies almost never report. Conversely, the small number of studies that deliberately tested delayed administration, including work comparing dosing at detorsion with dosing forty-five minutes later [87,88] and the ischemic postconditioning literature, which is defined by acting after the ischemic period [20], carry far more translational weight than their citation counts suggest. Any future systematic review of this field should stratify by timing before it stratifies by anything else.

### 6.3. Surrogate Endpoints and the Gradient of Evidence

As set out in Section 4, the endpoints that dominate the literature are the ones furthest from clinical relevance. A treatment effect on tissue malondialdehyde at four hours is, on its own, close to uninformative about whether a man will father a child twenty years later. The gradient matters because effect sizes shrink as endpoints become more meaningful. Agents that halve a biochemical marker typically improve the Johnsen score by a fraction of a point, and the handful of studies that carried through to sperm counts or to mating trials report correspondingly modest differences. This pattern is exactly what one would expect if the true clinical effect were real but small, and a small true effect is not a reason to abandon the idea. It is a reason to power a trial properly and to stop treating large biochemical effects as evidence of large clinical ones.

### 6.4. Risk of Bias and Publication Bias

Randomization, allocation concealment, blinded outcome assessment and a priori sample size calculation are reported in a minority of studies in this field, and sample sizes of six to eight animals per group are the norm. Small studies with unblinded histological scoring and no prespecified analysis plan generate inflated and unstable effect estimates. Beyond individual study quality lies the question of what has not been published. In experimental stroke, where the same problem was studied formally, systematic review and meta-analysis of a single agent demonstrated both substantial efficacy in the published animal literature and a clear signature of publication bias, followed by unambiguous clinical failure [143]. There is no reason to believe that the testicular literature is cleaner, and every reason to believe it is less clean, because it is dispersed across a larger number of smaller journals and has never been subjected to a formal risk of bias assessment using an instrument designed for animal studies. We should be explicit that the present review does not remedy that omission. A narrative review cannot substitute for a formal appraisal, and we have not applied a structured risk of bias instrument of the kind now available for animal research to the studies discussed here. The observations in this section and in Table 6 are therefore an informed characterization of the field rather than a measurement of it. A properly conducted systematic review with risk of bias assessment, stratified by timing of administration before anything else, is—in our view—among the most valuable next steps available and would require no laboratory work at all.

### 6.5. Lessons from Neuroprotection and Cardioprotection

The field of testicular ischemia–reperfusion is repeating, in almost every particular, an experiment that has already been run twice at far greater expense. In acute ischemic stroke, more than a thousand agents showed neuroprotection in animal models and essentially none translated. The most instructive case is the free radical trapping nitrone NXY-059, which was protective in rodent focal ischemia [144], satisfied the great majority of the consensus criteria that had been drawn up specifically to prevent translational failure, succeeded in a first large trial and then failed unambiguously in a second, and was subsequently found to offer no protection to human neurons in vitro [145]. Systematic comparison of preclinical, early phase and phase three studies in this field concluded that the mismatch arises from differences in the population studied, the timing of administration and the endpoints selected rather than from any single flaw [146,147].

Cardioprotection tells a similar story with one important difference. A very large number of agents reduced infarct size in animals and failed in patients [148,149]. But the one intervention in that field that has proved robust, ischemic conditioning, is procedural rather than pharmacological and acts on the reperfusion event itself. We think this is a signal worth taking seriously. In testicular torsion the equivalent intervention is available, costs nothing, and has never been tried in a human being.

## 7. The Human Evidence

### 7.1. What Exists in Testicular Torsion

It is worth stating the position in the plainest possible terms. We are not aware of a single adequately powered randomized controlled trial of any antioxidant, administered at or after surgical detorsion, with a fertility relevant primary endpoint, anywhere in the world literature. There is no licensed product for this indication. There is no guideline recommendation. Contemporary clinical reviews of testicular torsion continue to state, correctly, that surgical exploration is the only effective treatment and that no drug has been shown to help. What human data exist are observational and describe the natural history of the injury rather than any attempt to modify it, including the long-term functional deficits documented after anatomically successful salvage [9,10,26]. This is the central fact that the present review exists to draw attention to.

### 7.2. Indirect Evidence from Male Subfertility

The nearest human evidence comes from the use of oral antioxidants in subfertile men, and it is a cautionary rather than an encouraging body of work. The most recent Cochrane review included ninety randomized trials and more than ten thousand men, and found that antioxidants may increase live birth and clinical pregnancy rates, with the crucial qualifications that the certainty of the evidence was low to very low, that only a minority of trials reported live birth at all, and that the apparent benefit did not survive restriction of the analysis to studies at low risk of bias [21]. Systematic reviews and meta-analyses published subsequently have reached broadly similar conclusions, with modest apparent benefits on semen parameters and substantial methodological caveats [150]. Against this stands the largest well-conducted multicenter randomized placebo-controlled trial in the field, which used a combination formulation of vitamin C, vitamin E, selenium, l-carnitine, zinc, folic acid and lycopene and found no improvement in semen parameters, no improvement in sperm deoxyribonucleic acid integrity, and no improvement in pregnancy or live birth [22].

Two lessons follow. The first is that a positive meta-analysis of many small biased trials is not a substitute for one adequately powered trial, and that when the latter is finally performed it frequently disagrees with the former. The second is more specific to the pathology at hand. Chronic oral supplementation in a man with idiopathic oligozoospermia is a fundamentally different intervention from a single parenteral dose given at the moment of a defined reperfusion event, and the failure of the former tells us relatively little about the prospects of the latter. It would be a mistake, however, to convert that observation into optimism. The acute setting has genuine advantages, in that the mechanism is better defined, the timing is controlled, and the target is a discrete event rather than a diffuse chronic state. The subfertility experience nevertheless warns that such advantages have to be demonstrated in an adequately powered trial rather than inferred from them, and, as Section 2.1 noted, the residual injury available to be modified is itself likely to be modest.

### 7.3. Reductive Stress and the Limits of More Antioxidant

There is also a real possibility of harm, and it deserves more prominence than the experimental literature gives it. Reactive oxygen species are not simply toxins. They are obligatory signaling intermediates, and in the testis specifically they are required for spermatogonial stem cell self-renewal [56,57] and for the physiological capacitation of spermatozoa [59]. Excessive or prolonged antioxidant exposure shifts the redox environment towards a reductive state that perturbs disulfide bond formation, protamine-mediated chromatin condensation, mitochondrial bioenergetics and redox-sensitive transcription, and the nuclear factor erythroid 2-related factor 2 and Kelch-like ECH-associated protein 1 axis is now understood to respond to reductive as well as oxidative perturbation [151]. The most cited demonstration that high-dose antioxidant supplementation can cause net harm in humans remains the finding of increased prostate cancer incidence with long-term vitamin E in a very large prevention trial [152].

We do not think this argues against acute intervention in torsion. A single dose given over minutes to hours at a defined moment is pharmacologically remote from years of daily supplementation. It does argue very strongly against two things. The first is chronic postoperative antioxidant supplementation of the kind that is sometimes offered empirically after torsion surgery, for which there is no evidence and a plausible mechanism of harm. The second is the assumption, common in the experimental literature, that a larger reduction in tissue oxidant markers is necessarily a better outcome.

A specific caution attaches to the agent that we go on to recommend. Intravenous acetylcysteine is not an innocuous drug. Anaphylactoid reactions—which resemble true anaphylaxis clinically, with rash, pruritus, angioedema, bronchospasm and occasionally hypotension, but which are mediated non-immunologically—are well described from decades of use in paracetamol poisoning, and susceptibility is greater in females and in those with asthma or atopy [153]. In a pathway where the drug would be given to boys under general anesthesia, bronchospasm is the reaction that matters most, and a protocol would need an explicit management plan and a stopping rule for it. There is a second concern, which we raise because it follows from the argument of this subsection rather than because there is evidence for it. Acetylcysteine is a thiol reductant, and protamine-mediated chromatin condensation during spermiogenesis depends on disulfide bond formation, so the theoretical possibility that prolonged thiol exposure perturbs the very process whose disruption the treatment is meant to prevent should be examined preclinically before a 24 to 48 h human regimen is adopted.

### 7.4. The Specific Case of a Low Risk Antioxidant Such as Vitamin C

The argument of this review has been that the preclinical literature does not license clinical adoption. That conclusion concerns the strength of the evidence and the field as a whole, and it is worth separating from a narrower question that a reader is entitled to press. If an antioxidant is cheap, licensed, parenterally available and believed to be essentially harmless, does the absence of a positive trial really forbid giving it to a boy whose testis has just been reperfused? Ascorbate is the obvious test case. It is a physiological molecule, an established enzymatic cofactor and a chain breaking antioxidant in the aqueous phase, it is present in seminal plasma at concentrations well above those in serum, it is inexpensive, it has a long history of parenteral use, and the biological rationale in this setting is at least as strong as that of the more exotic compounds surveyed in Section 5. The case for permissive use runs as follows. The expected harm is very low, the expected benefit is uncertain but not negligible, the clinical event is unrepeatable, and the consequence of doing nothing may be a permanent functional deficit in a boy of twelve. On that reading, waiting for a trial that nobody is currently funding imposes a certain cost in foregone benefit in exchange for an uncertain gain in evidential rigor. We accept that this argument has real force, and we accept that our earlier formulation of the opposing position was more absolute than the evidence warrants.

Three considerations nevertheless lead us to keep ascorbate inside a research protocol rather than to endorse its empirical use, and we set them out so that a reader who weighs them differently can see exactly where the disagreement lies. The first is that the premise of harmlessness does not survive contact with the parenteral high dose literature. Vitamin C is close to harmless as a dietary constituent and at replacement doses. At the supraphysiological intravenous doses that would be required to influence a reperfusion burst it behaves as a different drug. Oxalate is a terminal metabolite of ascorbate, and oxalate nephropathy and nephrolithiasis are recognized complications of high dose infusion; haemolysis has been described in glucose 6 phosphate dehydrogenase deficiency, and interference with point of care glucometry is well documented [154]. More fundamentally, ascorbate at supraphysiological concentrations is not reliably an antioxidant. It reduces transition metals, and in the presence of the free iron liberated by ischemia and by heme breakdown it can drive Fenton chemistry and act as a pro-oxidant. This behavior has been characterized in the extracellular space above roughly five millimolar, and across that same concentration range its influence on neutrophil extracellular trap formation reverses direction, from inhibition at one millimolar and below to facilitation at five millimolar and above [155]. A reperfusing testis is precisely a compartment rich in free iron and infiltrating neutrophils, which is to say the compartment in which the pro-oxidant behavior of ascorbate is most likely to be expressed. Section 7.1 makes the same point in general terms about reactive oxygen species as physiological signaling intermediates, and ascorbate is a good illustration of why a molecule cannot be classified as protective independently of its concentration and its chemical environment.

The second consideration is empirical and it is the one we find most persuasive. The claim that a plausible, cheap and apparently safe antioxidant can be given without a trial because the downside is negligible has been tested directly in another acute condition, and it failed. In the LOVIT trial, eight hundred and seventy-two adults with sepsis receiving vasopressor therapy were randomized to intravenous vitamin C at fifty milligrams per kilogram every six hours for up to ninety-six hours or to matched placebo. The composite of death or persistent organ dysfunction at twenty-eight days occurred in one hundred and ninety-one of four hundred and twenty-nine patients given vitamin C, that is 44.5 percent, against one hundred and sixty-seven of four hundred and thirty-four given placebo, that is 38.5 percent, a risk ratio of 1.21 with a ninety-five percent confidence interval of 1.04 to 1.40 [156]. The signal was towards harm rather than benefit, it had not been anticipated by any prior evidence, and in fairness it did not persist in analyses adjusted for baseline covariates, which is a legitimate reason for caution in interpreting it. We do not claim that this result transfers to a single perioperative dose in an otherwise healthy adolescent, and the pharmacological and clinical settings differ in almost every respect. What it establishes is narrower and sufficient for our purposes. Intuitions about which antioxidants are harmless are not reliable, the class contains at least one agent whose apparent innocuousness did not survive a properly powered trial, and the agent in question is vitamin C itself. The vitamin E prostate cancer finding discussed in Section 7.3 makes the same point over a much longer timescale and with a different molecule [152]. Guidelines have since recommended against the routine use of intravenous vitamin C in adults with sepsis beyond standard nutritional supplementation, and have explicitly discouraged further small single-center trials of it [157].

The third consideration is that empirical use actively obstructs the trial that would settle the matter. If ascorbate becomes usual care at detorsion on the strength of its safety profile, the comparator disappears, the question becomes unanswerable, and a generation of boys receives an intervention of unknown effect. Something like this has already happened in this field on a smaller scale, since chronic postoperative antioxidant supplementation is offered empirically in some centers with no controlled evidence behind it at all. Our position, revised in the light of the foregoing, is therefore the following. We do not argue that administering vitamin C after detorsion would be unethical or reckless, and we withdraw any implication that the preclinical evidence positively contraindicates it. We argue that it is not yet justified as routine practice, and that the correct destination for a cheap, licensed and low risk candidate is the front of the trial queue rather than the back of the drug cupboard. On those criteria, ascorbate is a strong candidate for a first human study alongside the acetylcysteine and ischemic postconditioning arms proposed in Section 9. What would change our position is not a further rodent experiment. It is either a randomized comparison with a volumetric or spermatogenic endpoint, or a human dose finding study demonstrating that an achievable intravenous dose reaches the reperfusing testis at a concentration that is antioxidant rather than pro-oxidant in the presence of free iron. Both are feasible, neither has been done, and the second could be accomplished at a fraction of the cost of the trials this field has so far declined to run.

## 8. Anatomy of a Translational Failure

Bringing the preceding sections together, the failure to translate is not attributable to a single cause. It is the product of a set of mutually reinforcing barriers, each of which is individually surmountable. Table 7 sets them out alongside what we regard as the realistic mitigation for each. Three of them deserve emphasis because they are rarely acknowledged.

The first is structural rather than scientific. Testicular torsion is uncommon, the patients are children, the relevant outcome takes twenty years to mature, and the leading candidate molecules are off patent. There is therefore no commercial sponsor for a trial in this indication, and there never will be. Every successful trial in this field will have to be investigator-initiated, publicly or charitably funded, and run through a collaborative pediatric surgical network. Recognizing this changes what should be proposed, because it rules out expensive novel entities and favors cheap licensed drugs and procedural interventions.

The second is cultural. The experimental community has been rewarded for demonstrating that yet another compound reduces malondialdehyde in a rat testis, which is a publishable and inexpensive experiment, and has not been rewarded for the far harder work of replicating a single protocol across laboratories, of running an eight-week fertility endpoint, or of publishing a negative result. Until the incentives change, the catalog of effective molecules will continue to grow and the clinic will continue to look exactly as it does now.

The third is quantitative and follows from Section 2.1. Because outcome is dominated by the interval to reperfusion, and because effect sizes in this literature shrink as the endpoints become more meaningful, the absolute benefit available to a pharmacological adjunct is probably small. A small expected benefit is not an argument against doing the trial, but it is a decisive argument about which trial to do. It rules out an expensive agent, it demands realistic power rather than optimistic power, and it means that a network capable of delivering only one study in a decade has to weigh a drug trial against a trial of the pathway interventions that shorten the interval itself. We do not think the two compete for the same infrastructure in practice, but the comparison should be made explicitly rather than assumed away.

## 9. A Roadmap to First in Human Evaluation

Criticism is only useful if it produces a proposal. This section sets out what we believe a serious attempt at translation would look like, and it is deliberately conservative. Nothing in it requires a new molecule, a new technology or a large commercial investment.

### 9.1. Criteria for Selecting a Candidate

We propose six criteria. We regard them as necessary in principle, and Table 8 nevertheless retains several candidates that fail one or more of them, for reasons given at the end of this subsection. The agent must have demonstrated benefit in at least one study in which it was administered at or after the restoration of blood flow, in the sense defined in Section 5, rather than before torsion or during the ischemic interval. It must have a parenteral formulation, because oral administration is impractical in a fasted child about to receive a general anesthetic. It must already be licensed for human use, ideally in children, so that safety and dosing are established. It must be inexpensive and globally available, because torsion is a worldwide problem and the countries with the worst salvage rates are the ones least able to fund novel agents. It must have a plausible mechanism operating on the reperfusion phase rather than requiring transcriptional pre-arming. And it must have an acceptable interaction profile with routine anesthetic practice. Applying these filters to the interventions tabulated in this review reduces the field to a very small number, which is set out in Table 8. Two things should be said about that table before it is read. The shortlist is pragmatic rather than evidential, in that it ranks candidates by their fitness for an emergency pathway and not by the strength of the preclinical data behind them. And the entries that fail one or more criteria are retained deliberately, because they show where the obstacle lies, whether in formulation, in licensing or in the evidence itself, and not because we propose them for a trial. On our own reading, no candidate in the table yet has a preclinical base sufficient to justify a human study, and independent replication against a spermatogenic endpoint should precede any protocol.

### 9.2. Route and Timing

Three routes deserve consideration. Systemic intravenous administration at induction of anesthesia is the simplest and requires no change to surgical practice, but it delivers drug to an organ whose perfusion is by definition absent until the cord is untwisted, which means that the tissue concentration during the critical first minutes of reflow depends on how quickly the agent redistributes once flow is restored. Local infiltration of the spermatic cord, which surgeons already perform routinely with local anesthetic for postoperative analgesia, would deliver a high concentration directly into the vascular pedicle and could be given immediately before detorsion. Direct intratesticular or subtunical injection would achieve the highest local concentration but risks additional parenchymal injury and would be difficult to justify in a first study. Our view is that a first trial should use the intravenous route for simplicity and generalisability, with cord infiltration reserved for a subsequent dose-finding study.

On timing, the experimental data on the temporal profile of germ cell apoptosis argue for more than a single perioperative dose. Because apoptosis rises over hours and peaks around one day after repair [16,17], a regimen consisting of one intravenous dose at induction followed by continued administration over the first twenty-four to forty-eight postoperative hours is both mechanistically justified and entirely practical in a hospitalized patient.

### 9.3. Trial Architecture

The rate-limiting problem is not the drug but the numbers. Torsion is uncommon, and a single center performing thirty explorations a year cannot deliver a trial with a spermatogenic endpoint in any reasonable time. The only viable structure is a multicenter randomized trial embedded in an existing pediatric surgical or urological research network, with a small number of protocol steps so that recruitment can occur at any hour without specialist research staff. Randomization should be stratified by symptom duration, because that variable dominates outcome, and eligibility should be deliberately broad. Consent must use a deferred or waived model of the kind already accepted in pediatric emergency research, since obtaining considered written consent from a distressed family in the fifteen minutes before an emergency anesthetic is neither realistic nor ethical. That argument needs to be stated carefully, however, because it does not apply uniformly. A deferred model is defensible for the induction dose, which has to be given within minutes and cannot wait for a considered discussion. It is much harder to justify for the continued postoperative administration, for which there is ample time, and harder again in the older participants, many of whom are legally competent adults rather than children. We therefore propose a two-part model, in which the first dose is given under deferred or waived consent and continuation beyond the immediate perioperative period requires full prospective consent from the patient or from the person with parental responsibility. It follows that a trial spanning childhood and adulthood cannot be delivered by a pediatric network alone and will require adult urological collaboration.

We would argue for a staged approach. A feasibility and safety study of perhaps sixty patients would establish recruitment rates, protocol adherence, the practicality of the follow-up schedule, and the absence of harm. A subsequent efficacy trial should avoid a composite primary endpoint altogether. A composite that mixes a continuous volume measurement, a four-hormone profile, and a semen analysis available only in a postpubertal subset has no natural scoring rule, no defensible weighting and no clean handling of the missing component, and specifying one would reproduce precisely the endpoint looseness that Section 4 criticizes. We would therefore designate a single primary outcome, namely the ultrasonographic volume of the affected testis at twelve months, expressed in absolute terms and adjusted for age and body size, with the following as prespecified key secondary outcomes. These are the hormonal profile, including follicle stimulating hormone, luteinizing hormone, testosterone and inhibin B, and where the patient is postpubertal and consents, semen analysis with sperm deoxyribonucleic acid fragmentation. Absolute rather than relative volume is specified for the reason given in Section 2.4. Testicular volume is attractive as the primary outcome because it is objective, inexpensive, non-invasive, acceptable to adolescents and measurable in every participating center. Table 9 sets out a skeleton protocol.

The arithmetic of such a trial should be stated rather than left implicit, and it can be set out as a worked illustration even though the inputs will have to be confirmed against local data. If the standardized difference in twelve-month testicular volume between the arms is assumed to be half of a standard deviation, then approximately sixty-five participants per arm are required for eighty percent power at a two-sided alpha of five percent. If it is a quarter of a standard deviation, which is closer to what Section 6.3 leads us to expect, the requirement rises to approximately two hundred and fifty per arm. Suppose now that a participating center performs thirty explorations a year, that two thirds of those are randomized, and that three quarters of the randomized patients are retained to twelve months. Each center then contributes about fifteen evaluable participants a year, so a trial of one hundred and thirty patients in total is within reach of ten centers in a single year, whereas a trial of five hundred requires ten centers for more than three years or a substantially larger network. Deciding which of those effect sizes the field is prepared to pursue is therefore the first decision rather than the last, because it determines whether the study is feasible at all, and it is a decision that the present literature, with its uniformly large biochemical effects and its uniformly small histological ones, is poorly equipped to inform.

### 9.4. A Biomarker Strategy

A trial of this kind should also be designed to generate mechanistic information, because if it is negative the field needs to know whether the drug failed or the hypothesis failed. Three measurements are feasible without additional procedures. Peripheral venous blood taken at induction and at defined intervals after detorsion permits measurement of systemic oxidative stress markers and of the drug itself, giving both target engagement and pharmacokinetics. Where orchiectomy is performed the removed organ provides tissue for histology, immunohistochemistry and redox biochemistry, which converts an unavoidable clinical failure into a mechanistic dataset. And seminal oxidation-reduction potential measured at twelve months in postpubertal participants provides a downstream redox readout that has already been shown to differ in men with a history of torsion [10,82].

## 10. Emerging Directions

Several developments could change the calculus over the next decade. The first is subcellular targeting. Conventional antioxidants distribute according to their physicochemical properties rather than according to where the oxidants are made, which is a fundamental inefficiency given that much of the burst originates inside mitochondria. Lipophilic cation-conjugated antioxidants accumulate several hundredfold within the mitochondrial matrix and have shown protection in other models of ischemia and reperfusion through nuclear factor erythroid 2-related factor 2-dependent mechanisms [158]. No study has yet applied this class properly to the testis.

The second is death pathway specificity. If ferroptosis contributes materially to germ cell and Sertoli cell loss after reperfusion [72,73], then agents that inhibit lipid peroxide propagation or support glutathione peroxidase 4 are conceptually distinct from general scavengers and may succeed where the latter have not. The same reasoning applies to selective inflammasome inhibition, which is now entering clinical development in other indications. The third is formulation science. Nanoparticle and liposomal carriers have been used to deliver neuroprotective agents with controlled release in models of cerebral ischemia [159], and the same approach could in principle convert a promising but currently unusable compound such as astaxanthin into a parenteral agent suitable for emergency use.

The fourth, and in our judgment the most important, is methodological rather than chemical. A coordinated preclinical replication program, in which two or three lead interventions are tested against a single agreed protocol in three or four independent laboratories, with prespecified spermatogenic endpoints, blinded assessment and publication of results regardless of direction, would tell the field more than another two hundred single-center studies of novel plant extracts. Such programs now exist in stroke and in cardiology. There is no reason one could not exist here, and it would be considerably cheaper.

## 11. Conclusions

Testicular torsion is one of the very few clinical situations in which the moment of reperfusion is created deliberately, at a known time, by a clinician, in a controlled environment. The mechanism is understood in considerable molecular detail, the window for intervention probably extends well beyond the operation itself, since germ cell death in rodents peaks approximately one day after repair, and a large experimental literature has shown that the injury can be attenuated pharmacologically in animals. Despite this, no antioxidant is given as part of routine management in any guideline we have been able to identify, and no properly designed trial has asked whether one should be. The gap is not in the hypothesis. It lies in how the evidence was built, and in the absence of any commercial sponsor to fund the studies that would settle the question.

The remedy is correspondingly specific. Candidate selection should be restricted to agents that work when given after detorsion, that have a parenteral formulation, and that are already licensed and inexpensive. The resulting shortlist should be replicated across laboratories against spermatogenic endpoints rather than four-hour biochemistry. The first human study should then be a modest, investigator-initiated, multicenter feasibility trial. On present evidence, the two interventions that best satisfy these criteria are N-acetylcysteine, which is inexpensive, intravenous, licensed in children and mechanistically upstream, and ischemic postconditioning, which requires no medicinal product at all, although its protocol still has to be defined in an animal model before it can be taken to patients. Neither is glamorous. Both are testable. Two things follow for practice today. Until a trial has been done, we do not think any antioxidant should be adopted as routine practice at or after detorsion, and chronic postoperative supplementation in particular has no evidential basis and a plausible mechanism of harm. And the next step is not the trial itself. It is the unglamorous work that has to precede it, namely a systematic review stratified by timing of administration, a coordinated preclinical replication of one or two candidates against spermatogenic endpoints, and a characterization of the human time course of germ cell death after detorsion. After four decades of rodent experiments, testability is the property that matters most, and testability has to be earned before it can be exercised.

## Figures and Tables

**Table 1:** Principal sources of reactive species during testicular ischemia and reperfusion, their approximate temporal windows and the corresponding pharmacological targets.

Source	Trigger and Approximate Timing	Principal Species Generated	Representative Targeting Strategy
Xanthine oxidase	Xanthine dehydrogenase converted during ischemia, activated within seconds of oxygen re-entry	Superoxide anion, hydrogen peroxide	Allopurinol, febuxostat
Mitochondrial reverse electron transport	Succinate accumulated during ischemia oxidized at complex I in the first minutes of reflow	Superoxide anion at complex I	Coenzyme Q10, mitochondria-targeted antioxidants
NADPH oxidase family (NOX2, NOX4, NOX5)	Endothelial, peritubular and leukocyte activation, minutes to hours	Superoxide anion	Apocynin, diphenyleneiodonium, NOX-selective inhibitors
Infiltrating neutrophils and myeloperoxidase	Adhesion to subtunical venules from about one hour, peak over four to twenty four hours	Hypochlorous acid, chloramines, superoxide anion	Anti-adhesion strategies, colchicine, cyclooxygenase inhibitors
Uncoupled and inducible nitric oxide synthase	Transcriptional induction over hours after reflow	Nitric oxide, peroxynitrite	Aminoguanidine, propofol, selective iNOS inhibition
Labile iron and phospholipid peroxidation	Iron released from damaged proteins, hours after reflow	Lipid hydroperoxides, four-hydroxynonenal	Iron chelation, GPX4 support, Nrf2 activators
Exhaustion of endogenous defenses	Progressive consumption of SOD, catalase, GPx and glutathione throughout reperfusion	Loss of buffering capacity rather than new species	N-acetylcysteine, alpha lipoic acid, selenium, Nrf2 activators

**Table 2:** Endpoint families used in testicular ischemia–reperfusion research, ranked by proximity to the outcome that matters clinically. The frequency column records our qualitative impression of the literature we examined and is not the result of a formal count.

Endpoint Family	Representative Measures	Typical Time Point	Proximity to Patient-Relevant Outcome	Frequency in the Literature (Qualitative)
Tissue redox chemistry	MDA, SOD, catalase, GPx, GSH, TAS, TOS, MPO	1 to 24 h after detorsion	Low. Confirms that a reducing agent reached the tissue	Very high
Molecular cell death signaling	TUNEL, caspase 3, Bax to Bcl-2 ratio, NLRP3, IL-1β, TNF-α	4 to 24 h after detorsion	Low to moderate. Mechanistically informative	High
Histomorphometry	Johnsen score, tubule diameter, germinal epithelial height	24 h to 8 weeks	Moderate to high. Directly reflects spermatogenic integrity	Moderate
Gametic function	Epididymal sperm count, motility, morphology, DNA fragmentation	At least one spermatogenic cycle	High	Low
Endocrine function	Intratesticular and serum testosterone, LH, FSH, inhibin B	2 to 8 weeks	High	Low
Fertility	Mating trials, pregnancy rate, litter size	8 weeks or more	Highest	Very low
Human translational	Semen analysis, DNA fragmentation, seminal ORP, testicular volume, hormone profile	6 to 12 months after surgery	Definitive	Absent from interventional studies

**Table 6:** Methodological features of the experimental literature on antioxidants in testicular ischemia–reperfusion injury and their consequences for translation.

Feature	Typical Practice in the Field	Consequence	What Should Be Done Instead
Species and age	Adult rat predominant, prepubertal animals uncommon	Poor match to the peripubertal human patient	Prepubertal or peripubertal animals as the default
Torsion angle and duration	540 to 1080 degrees, 30 min to 8 h, rarely justified	Studies not comparable, pooling invalid	A small number of standardized protocols agreed across laboratories
Timing of administration	Recoverable from the report for only 18 of the 53 tabulated interventions, and before torsion in 5 of those 18	Answers a prophylaxis question that has no clinical counterpart, and is more often simply unrecoverable	Timing stated explicitly relative to the restoration of flow, and administration at or after that point for any agent intended for clinical use
Reperfusion interval	Commonly 1 to 24 h	Captures biochemistry, misses spermatogenic outcome	At least one full spermatogenic cycle for the primary endpoint
Primary endpoint	Tissue MDA and antioxidant enzyme activity	Large effects with little clinical meaning	Histomorphometry and gametic function as primary, chemistry as secondary
Group size	Six to eight animals, sample size rarely calculated	Inflated and unstable effect estimates	A priori power calculation against a histological endpoint
Randomization and blinding	Reported in a minority of studies	Systematic overestimation of benefit	Randomization, allocation concealment and blinded scoring reported explicitly
Dose selection	Single dose, seldom justified, ranges over orders of magnitude	No basis for human dose selection	Dose ranging with a pharmacokinetic rationale
Negative results	Rarely published	Apparent universal efficacy	Preregistration of animal protocols and publication of negative findings
Replication	Almost no independent replication of a specific protocol	Corroboration is illusory	Multicenter preclinical studies for lead candidates

**Table 7:** Barriers to clinical translation of antioxidant therapy in testicular torsion and realistic mitigations.

Domain	Barrier	Realistic Mitigation
Preclinical design	Dominance of pretreatment protocols with no clinical counterpart	Restrict candidate selection to agents effective when given at or after detorsion
Preclinical design	Endpoints dominated by short term tissue chemistry	Require a spermatogenic or gametic primary endpoint before human testing
Preclinical design	Absent replication and probable publication bias	Multicenter preclinical replication of two or three leads with preregistered protocols
Pharmacology	Many leads are poorly bioavailable plant compounds without parenteral formulations	Prioritize licensed drugs with existing intravenous formulations
Pharmacology	Dose selection in animals spans orders of magnitude with no human basis	Pharmacokinetic bridging studies and allometric dose justification
Clinical logistics	Intervention must be deliverable within minutes at any hour in any hospital	Single dose intravenous agent given at induction of anesthesia
Trial population	Uncommon condition, heterogeneous ischemic time, no reliable pre-randomization viability measure	Multicenter network, stratification by symptom duration, generous eligibility
Endpoints	The outcome of real interest matures over decades	Composite of testicular volume, hormonal profile and semen analysis at 12 months as a validated intermediate
Ethics and consent	Pediatric emergency setting, no time for considered consent	Deferred or waived consent frameworks already used in pediatric emergency research
Funding	No commercial sponsor for an off patent drug in a rare pediatric indication	Investigator initiated design, public and charitable funding, adoption by a surgical trials network
Academic incentives	Positive small studies of novel compounds are cheap and publishable	Journals and funders to require timing, blinding and endpoint standards for this model

**Table 8:** Candidate agents ranked against the six criteria proposed for first-in-human evaluation. Entries that fail one or more criteria are retained in order to show where the obstacle lies and are not proposed for a trial.

Candidate	Effective When Given at or After Restoration of Flow	Parenteral Formulation	Licensed in Children	Cost and Availability	Overall Assessment
N-acetylcysteine	Yes	Yes, well established intravenous regimens	Yes, extensive pediatric experience	Very low, globally available	Strongest pragmatic candidate
Ischemic postconditioning	Yes by definition	Not applicable, procedural	Not applicable	No cost	Strongest procedural candidate, testable immediately
Melatonin	Yes in several studies	Available in some jurisdictions	Yes, oral, in pediatric sleep disorders	Low	Strong candidate limited by formulation availability
Dexmedetomidine	Yes	Yes	Yes, pediatric anesthesia	Moderate	Attractive because it can be incorporated into the anesthetic
Erythropoietin	Yes	Yes	Yes	Moderate to high	Plausible but thrombotic risk requires justification
Astaxanthin	Yes, including delayed dosing	No approved parenteral form	No	Low as a supplement	Mechanistically attractive, formulation is the obstacle
Hydrogen rich saline	Yes	Yes, investigational	No	Low	Promising but regulatory pathway unclear
Polyphenols as a class	Variable	Generally no	No	Low	Not currently translatable despite the volume of preclinical data

**Table 9:** Skeleton protocol for a first in human study of an antioxidant adjunct at the time of surgical detorsion.

Protocol Element	Proposal	Rationale
Design	Multicenter, randomized, double blind, placebo controlled, two stage	Only structure capable of recruiting adequate numbers in an uncommon condition
Population	Males aged 8 to 25 years undergoing emergency scrotal exploration for suspected testicular torsion. Neonatal torsion excluded	Captures the peripubertal and young adult incidence peak and reflects real emergency practice. Neonatal torsion is excluded because it is usually prenatal and already established at birth, so that no reperfusion event remains to be targeted
Eligibility	Broad. No exclusion by symptom duration or by predicted viability	Viability cannot be predicted reliably before exploration
Stratification	Symptom duration under 6 h, 6 to 12 h, over 12 h	Symptom duration is the dominant prognostic variable
Intervention	Single intravenous dose at induction followed by administration over the first 24 to 48 postoperative hours	Matches the temporal profile of germ cell apoptosis after repair
Comparator	Matched placebo, all other care unchanged	Preserves equipoise and blinding
Consent	Deferred or waived consent for the induction dose only, with full prospective consent required before continued postoperative administration	Established in pediatric emergency research for the time critical dose, while the postoperative doses allow time for proper consent, including from participants who are competent adults
Stage 1 endpoint	Feasibility, recruitment rate, protocol adherence, safety	Determines whether the definitive trial is deliverable
Stage 2 primary endpoint	Absolute ultrasonographic volume of the affected testis at 12 months, adjusted for age and body size	Single and objective, measurable in all centers, and absolute rather than relative because contralateral injury or protection would blunt a ratio. Powering is worked through in Section 9.3
Secondary endpoints	Gonadotropin, testosterone and inhibin B profile, semen analysis where applicable, orchiectomy rate, sperm DNA fragmentation, seminal oxidation reduction potential, patient reported outcomes	Captures endocrine, gametic, clinical, molecular and experiential dimensions without loading them into the primary outcome
Mechanistic substudy	Serial plasma redox markers and drug concentrations, tissue analysis of orchiectomy specimens	Distinguishes failure of the drug from failure of the hypothesis
Governance	Investigator initiated, delivered through a pediatric surgical trials network, publicly or charitably funded	No commercial sponsor will fund an off patent drug in this indication

## Data Availability

No new data were created or analyzed in this study. Data sharing is not applicable to this article.

## Abbreviations

The following abbreviations are used in this manuscript:

AKT	Protein kinase B
AMPK	Adenosine monophosphate-activated protein kinase
ATP	Adenosine triphosphate
Bax	Bcl-2-associated X protein
Bcl-2	B-cell lymphoma 2
DNA	Deoxyribonucleic acid
eNOS	Endothelial nitric oxide synthase
FSH	Follicle-stimulating hormone
GPx	Glutathione peroxidase
GPX4	Glutathione peroxidase 4
GSH	Reduced glutathione
HO-1	Heme oxygenase 1
IL	Interleukin
IL-1β	Interleukin 1 beta
iNOS	Inducible nitric oxide synthase
IV	Intravenous
LH	Luteinizing hormone
MAPK	Mitogen-activated protein kinase
MDA	Malondialdehyde
MMP	Matrix metalloproteinase
MPO	Myeloperoxidase
NADPH	Nicotinamide adenine dinucleotide phosphate
NLRP3	NOD-like receptor family pyrin domain containing 3
NOX	NADPH oxidase
Nrf2	Nuclear factor erythroid 2-related factor 2
ORP	Oxidation-reduction potential
PI3K	Phosphoinositide 3-kinase
ROS	Reactive oxygen species
SAFE	Survivor activating factor enhancement
SOD	Superoxide dismutase
TAS	Total antioxidant status
TNF-α	Tumor necrosis factor alpha
TOS	Total oxidant status
TUNEL	Terminal deoxynucleotidyl transferase-mediated dUTP nick end labeling
TXNIP	Thioredoxin interacting protein
XDH	Xanthine dehydrogenase
XO	Xanthine oxidase
